# Supplementary material for: Sociodemographic, labour market marginalisation and medical characteristics as risk factors for reinfarction and mortality within 1 year after a first acute myocardial infarction: a register-based cohort study of a working age population in Sweden
Source: BMJ Open. 2019 Dec 18;9(12):e033616. doi: 10.1136/bmjopen-2019-033616 (PMC6937026; doi:10.1136/bmjopen-2019-033616)
Supplement: Supplementary data [file bmjopen-2019-033616supp001.pdf]

Supplementary Table

Table. Crude and adjusted hazard ratios (HR) and 95% Confidence Interval (CI) for mortality due to circulatory disorders during first year of follow-up in individuals with diagnosis of acute myocardial infarction (AMI) from inpatient care in 2008-2010 in Sweden (N=15 069)

Characteristics of patients with AMI	**Mortality (First year)** n (%)	Crude model	Model 1[a]	Model 2[b]	Model 3[c]
		HR (95% CI)			
**Socio-demographic characteristics[d]**					
Sex					
Men	365 (3.1)	1	1	1	1
Women	133 (3.6)	1.14 (0.94-1.40)	0.92 (0.75-1.13)	0.91 (0.74-1.12)	0.93 (0.75-1.15)
Age					
25-45	24 (1.7)	1	1	1	1
46-55	99 (2.1)	1.21 (0.77-1.89)	1.04 (0.66-1.63)	1.02 (0.65-1.60)	1.15 (0.73-1.81)
56-64	375 (4.1)	2.40 (1.59-3.63)	1.89 (1.24-2.90)	1.85 (1.21-2.84)	2.10 (1.37-3.23)
Education (years)[e]					
Compulsory (≤9)	190 (4.1)	1.61 (1.25-2.09)	1.26 (0.96-1.64)	1.25 (0.96-1.63)	1.23 (0.94-1.61)
High school (10–12)	225 (3.0)	1.16 (0.90-1.49)	1.02 (0.79-1.32)	1.02 (0.79-1.31)	1.02 (0.79-1.32)
University (>12)	83 (2.6)	1	1	1	1
Country of birth[f]					
Sweden	408 (3.3)	1	1	1	1
Other Nordic countries	44 (5.0)	1.51 (1.11-2.07)	1.20 (0.87-1.64)	1.20 (0.87-1.64)	1.32 (0.97-1.81)
Europe (except Nordic countries)	11 (2.5)	0.73 (0.40-1.32)	0.69 (0.38-1.25)	0.70 (0.38-1.28)	0.76 (0.42-1.39)
Non-European countries	35 (2.1)	0.61 (0.43-0.87)	0.62 (0.43-0.89)	0.63 (0.44-0.90)	0.67 (0.46-0.96)
Type of living area[g]					
Big cities	150 (3.2)	1	1	1	1
Medium sized cities	173 (3.2)	0.99 (0.79-1.23)	0.92 (0.74-1.15)	0.93 (0.75-1.17)	0.97 (0.78-1.21)
Small towns/villages	175 (3.3)	1.04 (0.83-1.29)	0.92 (0.73-1.15)	0.94 (0.75-1.18)	0.93 (0.74-1.16)
Family situation[h]					
Married[i] living without children	172 (3.4)	1.95 (1.48-2.57)	1.41 (1.06-1.88)	1.40 (1.05-1.87)	1.47 (1.10-1.97)

Married[i] living with children	72 (1.8)	1	1	1	1
Single[j] living without children	231 (4.2)	2.40 (1.84-3.12)	1.64 (1.24-2.15)	1.59 (1.20-2.09)	1.61 (1.22-2.12)
Single[j] living with children	23 (2.8)	1.60 (1.00-2.56)	1.36 (0.84-2.18)	1.33 (0.83-2.15)	1.37 (0.85-2.20)
**Work-related characteristics**					
Trajectory groups of SA/DP [*] ***three years before and at inclusion***					
Low increasing	210 (2.6)	1	1	1	1
Constant low	47 (1.7)	0.66 (0.48-0.90)	0.65 (0.47-0.89)	0.64 (0.46-0.88)	0.58 (0.42-0.80)
Middle increasing	47 (3.2)	1.25 (0.91-1.72)	1.22 (0.89-1.68)	1.17 (0.85-1.62)	0.93 (0.67-1.29)
High decreasing	13 (1.5)	0.60 (0.34-1.05)	0.47 (0.26-0.82)	0.44 (0.25-0.77)	0.33 (0.18-0.58)
Constant high	181 (8.3)	3.29 (2.69-4.01)	3.06 (2.47-3.80)	2.82 (2.23-3.57)	1.97 (1.54-2.53)
Unemployment in the year before inclusion					
No unemployment	462 (3.3)	1	1	1	1
1-180 days	18 (2.1)	0.63 (0.39-1.01)	0.95 (0.59-1.54)	0.94 (0.58-1.52)	0.94 (0.58-1.52)
>180 days	18 (4.3)	1.30 (0.81-2.08)	1.82 (1.13-2.93)	1.83 (1.14-2.96)	1.83 (1.13-2.97)
**AMI characteristics**					
Inpatient care due to AMI (median 4 days) [k] ***at inclusion***					
1-4 days	316 (3.3)	1	1	1	1
>4 days	182 (3.2)	0.95 (0.79-1.14)	0.86 (0.72-1.04)	0.86 (0.71-1.03)	0.74 (0.61-0.89)
Type of infarction [k] ***at inclusion***					
STEMI[l]	219 (4.1)	2.78 (2.20-3.51)	2.98 (2.35-3.76)	3.00 (2.37-3.80)	4.29 (3.36-5.48)
Non-STEMI[m]	104 (1.5)	1	1	1	1
Unspecified	175 (5.5)	3.73 (2.93-4.75)	3.62 (2.84-4.61)	3.58 (2.81-4.57)	3.13 (2.45-4.00)
Coronary revascularisation characteristics [n] ***at inclusion***					
Percutaneous coronary intervention	211 (2.0)	1	1	1	1
Coronary artery bypass grafting	10 (2.9)	1.47 (0.78-2.78)	1.36 (0.72-2.57)	1.38 (0.73-2.60)	1.88 (0.99-3.56)
Others	277 (6.1)	3.12 (2.61-3.74)	2.80 (2.33-3.36)	2.76 (2.29-3.32)	3.37 (2.76-4.11)

Morbidity characteristics					
**Somatic (co)-morbidities[o]**					
**three years before and/or at inclusion**					
Musculoskeletal disorders					
No	423 (3.3)	1	1	1	1
Yes	75 (3.2)	0.98 (0.77-1.26)	0.84 (0.66-1.08)	0.84 (0.66-1.08)	0.91 (0.71-1.18)
Diabetes mellitus[p]					
No	360 (2.8)	1	1	1	1
Yes	138 (5.2)	1.87 (1.53-2.27)	1.55 (1.27-1.89)	1.56 (1.27-1.91)	1.53 (1.24-1.90)
Renal insufficiency					
No	458 (3.0)	1	1	1	1
Yes	40 (15.4)	5.29 (3.83-7.30)	3.88 (2.78-5.40)	3.75 (2.68-5.25)	2.75 (1.94-3.89)
Hypertension					
No	353 (3.5)	1	1	1	1
Yes	145 (2.8)	0.79 (0.65-0.96)	0.71 (0.59-0.87)	0.71 (0.59-0.87)	0.67 (0.55-0.82)
Stroke					
No	477 (3.1)	1	1	1	1
Yes	21 (10.1)	3.33 (2.15-5.15)	2.26 (1.45-3.51)	2.30 (1.48-3.58)	1.96 (1.25-3.06)
Cancer					
No	459 (3.2)	1	1	1	1
Yes	39 (4.1)	1.28 (0.93-1.78)	1.15 (0.83-1.60)	1.13 (0.81-1.57)	1.01 (0.72-1.41)
Other somatic disorders					
No	115 (2.3)	1	1	1	1
Yes	383 (3.7)	1.62 (1.32-2.00)	1.43 (1.16-1.77)	1.41 (1.14-1.75)	1.36 (1.10-1.70)
**Mental co-morbidities**					
Common mental disorders[o]					
three years before and/or at inclusion					
No	471 (3.2)	1	1	–	1
Yes	27 (3.4)	1.04 (0.70-1.53)	0.76 (0.51-1.13)	–	0.81 (0.54-1.21)
Other mental disorders[o]					

three years before and/or at inclusion					
No	431 (3.1)	1	1	–	1
Yes	67 (5.0)	1.63 (1.26-2.11)	1.31 (1.01-1.71)	–	1.28 (0.98-1.68)
Antidepressants in the year before inclusion					
No antidepressants	418 (3.1)	1	1	–	1
Small doses (<0.5 DDD[q])	24 (4.0)	1.26 (0.84-1.91)	1.05 (0.69-1.59)	–	0.96 (0.63-1.46)
Moderate doses (0.5-1.5 DDD)	42 (4.8)	1.57 (1.14-2.16)	1.15 (0.83-1.59)	–	1.17 (0.84-1.63)
High doses (>1.5 DDD)	14 (3.4)	1.12 (0.66-1.90)	0.70 (0.41-1.21)	–	0.81 (0.47-1.40)
Anxiolytics in the year before inclusion					
No Anxiolytics	431 (3.1)	1	1	–	1
Small doses (<0.5 DDD)	45 (4.5)	1.48 (1.09-2.01)	1.10 (0.80-1.51)	–	1.06 (0.77-1.46)
Moderate doses (0.5-1.5 DDD)	13 (6.1)	1.98 (1.14-3.44)	1.10 (0.63-1.93)	–	1.04 (0.59-1.83)
High doses (>1.5 DDD)	<10 (8.0)	2.63 (1.36-5.09)	1.15 (0.59-2.25)	–	1.22 (0.62-2.40)
Sedatives in the year before inclusion					
No sedatives	394 (2.9)	1	1	–	1
Small doses (<0.5 DDD)	41 (4.5)	1.58 (1.14-2.17)	1.33 (0.96-1.85)	–	1.16 (0.83-1.61)
Moderate doses (0.5-1.5 DDD)	40 (6.5)	2.25 (1.63-3.12)	1.42 (1.01-1.99)	–	1.28 (0.90-1.80)
High doses (>1.5 DDD)	23 (7.5)	2.65 (1.74-4.03)	1.52 (0.98-2.36)	–	1.47 (0.95-2.28)

[a] Adjusted for sex, age, educational level, country of birth, type of living area, family situation, trajectory groups of SA/DP and previous unemployment

[b] Adjusted for sex, age, educational level, country of birth, type of living area, family situation, trajectory groups of SA/DP and previous unemployment, in- and specialised outpatient care due to common mental disorders and other mental disorders, antidepressants, anxiolytics and sedatives; Mental comorbidities were not mutually controlled

[c] Adjusted for sex, age, educational level, country of birth, type of living area, family situation, trajectory groups of SA/DP and previous unemployment, in- and specialised outpatient care due to common mental disorders and other mental disorders, antidepressants, anxiolytics, sedatives, inpatient days due to AMI, type of infarction, musculoskeletal disorders, diabetes mellitus, renal insufficiency, hypertension, stroke, cancer and other somatic disorders; Mental comorbidities were not mutually controlled

[d] Measured on 31st December of the year preceding acute myocardial infarction

[e] Missing data is considered compulsory education
[f] Missing data is considered Non-European countries
[g] Type of living area: big cities (Stockholm, Gothenburg and Malmö); medium sized cities (cities with more than 90 000 inhabitants within 30 km distance from the centre of the city); small cities/villages/rural
[h] Missing data is considered single living without children
[i] Married includes all living with partner; cohabitant
[j] Single includes divorced, separated, or widowed
[k] See method section for the International Classification of Diseases version 10 (ICD-10) codes or the Anatomic Therapeutic Chemical classification system (ATC) codes
[l] ST-elevation myocardial infarction
[m] Non-ST-elevation myocardial infarction
[n] See method section for the Classification of Surgical Procedures
[o] Measured by main or side diagnosis in in- or specialised outpatient care
[p] Additionally measured by prescribed antidiabetic medication
[q] Daily dispensed dose (DDD)
