# Supplementary material for: Sociodemographic, labour market marginalisation and medical characteristics as risk factors for reinfarction and mortality within 1 year after a first acute myocardial infarction: a register-based cohort study of a working age population in Sweden
Source: BMJ Open. 2019 Dec 18;9(12):e033616. doi: 10.1136/bmjopen-2019-033616 (PMC6937026; doi:10.1136/bmjopen-2019-033616)
Supplement: Author's manuscript [file bmjopen-2019-033616.draft_revisions.pdf]

BMJ Open is committed to open peer review. As part of this commitment we make the peer review history of every article we publish publicly available.

When an article is published we post the peer reviewers' comments and the authors' responses online. We also post the versions of the paper that were used during peer review. These are the versions that the peer review comments apply to.

The versions of the paper that follow are the versions that were submitted during the peer review process. They are not the versions of record or the final published versions. They should not be cited or distributed as the published version of this manuscript.

BMJ Open is an open access journal and the full, final, typeset and author-corrected version of record of the manuscript is available on our site with no access controls, subscription charges or pay-per-view fees (http://bmjopen.bmj.com).

If you have any questions on BMJ Open's open peer review process please email info.bmjopen@bmj.com

BMJ Open

Socio-demographic, labour market marginalisation, and medical characteristics as risk factors for re-infarction and mortality within one year after a first acute myocardial infarction-A register-based cohort study of a working age population in Sweden

Journal:	*BMJ Open*
Manuscript ID	bmjopen-2019-033616
Article Type:	Research
Date Submitted by the Author:	13-Aug-2019
Complete List of Authors:	Wang, Mo; Karolinska Institute,<br>Vaez, Marjan; Karolinska Institute, Division of Insurance Medicine, Department of Clinical Neuroscience<br>Dorner, Thomas; Medizinische Universitat Wien, Centre for Public Health, Institute of Social Medicine<br>Rahman, Syed; Karolinska Institute, Division of Insurance Medicine, Department of Clinical Neuroscience<br>Helgesson, Magnus; Karolinska Institute, Division of Insurance Medicine, Department of Clinical Neuroscience<br>Ivert, Torbjörn; Karolinska Institute, Department of Molecular Medicine and Surgery<br>Mittendorfer-Rutz, Ellenor; Karolinska Institute, Division of Insurance Medicine, Department of Clinical Neuroscience
Keywords:	Acute myocardial infarction, Re-infarction, Mortality, Sick leave, Disability pension, Insurance Medicine

SCHOLARONE™
Manuscripts

Risk factors for re-infarction and mortality after acute myocardial infarction

Socio-demographic, labour market marginalisation, and medical characteristics as risk factors for re-infarction and mortality within one year after a first acute myocardial infarction-A register-based cohort study of a working age population in Sweden

Mo Wang[1], Marjan Vaez[1], Thomas Dorner[2], Syed Rahman[1], Magnus Helgesson[1], Torbjörn Ivert[3], Ellenor Mittendorfer-Rutz[1]

[1] Division of Insurance Medicine, Department of Clinical Neuroscience, Karolinska Institutet, SE 171 77 Stockholm, Sweden

[2] Department of Social and Preventive Medicine, Centre for Public Health, Medical University of Vienna, Vienna, Austria

[3] Department of Molecular Medicine and Surgery, Karolinska Institutet and Heart and Vascular Theme, Karolinska University Hospital, Stockholm, Sweden

Send requests for offprint to Corresponding author:

Mo Wang

Department of Clinical Neuroscience

Division of Insurance Medicine

Karolinska Institutet

SE-171 77 Stockholm

Sweden

E-mail: mo.wang@ki.se

Tel.: +46-8-524 832 24

Fax: +46-8-524 832 05

Word count: 3178

For peer review only - http://bmjopen.bmj.com/site/about/guidelines.xhtml

Risk factors for re-infarction and mortality after acute myocardial infarction

Abstract

Objectives: Research covering a wide range of risk factors related to the prognosis during the first year after an acute myocardial infarction (AMI) is insufficient. This study aimed to investigate whether socio-demographic, labour market marginalisation, and medical characteristics before/at AMI were associated with subsequent re-infarction and all-cause mortality.

Design: Population-based cohort study.

Participants: The cohort included 15 069 individuals aged 25-64 years who had a first AMI during 2008-2010.

Primary and secondary outcome measures: The outcome measures consisted of re-infarction and all-cause mortality within one year following an AMI, which were estimated by univariate and multivariable hazard ratios (HR) and 95% confidence intervals (CI) by Cox regression.

Results: Socio-demographic characteristics such as lower education showed a 1.1- and 1.3-fold higher risk for re-infarction and mortality, respectively. Older age was associated with a higher risk of mortality while being born in non-European countries showed a lower risk of mortality. Labour market marginalisation such as previous long-term work disability was associated with a 2-fold higher risk of mortality. Regarding medical characteristics, ST-elevation myocardial infarction was predictive for re-infarction (HR: 1.14, 95% CI: 1.07-1.21) and all-cause mortality (HR: 3.80, 95% CI: 3.08-4.68). Moreover, diabetes mellitus, renal insufficiency, stroke, cancer, and mental disorders were associated with a higher risk of mortality (range of HRs: 1.24-2.59).

Conclusions: Socio-demographic and medical risk factors were identified as risk factors for mortality and re-infarction after AMI, including older age, immigration status, somatic and mental co-morbidities. Previous long-term work disability and infarction type provide useful

For peer review only

For peer review only - http://bmjopen.bmj.com/site/about/guidelines.xhtml

information for predicting adverse outcomes after AMI during the first year, particularly for mortality.

Keywords: Acute myocardial infarction; Re-infarction; Mortality; Sick leave; Disability pension; Insurance Medicine.

For peer review only

For peer review only - http://bmjopen.bmj.com/site/about/guidelines.xhtml

Risk factors for re-infarction and mortality after acute myocardial infarction

ARTICLE SUMMARY

Strengths and limitations of this study

- This is a population-based cohort study on all patients with acute myocardial infarction from inpatient care.
- The Swedish national-wide register data has high quality, which reduces the risk of recall bias regarding exposure and outcome.
- Despite a wide range of risk factors that have been examined, some potential for residual confounding by unmeasured factors remains.
- There is no available information on sick-leave spells that are shorter than 14 days among employed individuals.

For peer review only

For peer review only - http://bmjopen.bmj.com/site/about/guidelines.xhtml

Risk factors for re-infarction and mortality after acute myocardial infarction

INTRODUCTION

Acute myocardial infarction (AMI), is the leading cause of mortality worldwide and re-infarction is common, ranging from 8% to 20% in the first year (1). Over the past decade, percutaneous coronary intervention (PCI) and medication have reduced mortality in AMI patients (2, 3). Despite this progress, AMI remains a major cause of mortality and disability. For patients who survive a first AMI, post-discharge optimal medical management and healthy life-style are essential. Particularly, re-infarction and heart failure can occur after an AMI, influencing quality of life and increasing healthcare costs (1). Knowledge of risk factors for re-infarction and mortality in the first year after an AMI could improve the ability of healthcare providers to reduce progression of disease as well as improve survival after AMI.

Previous studies have reported risk factors for re-infarction and mortality in patients with AMI, mainly focusing on events within the first month after discharge (4). Socio-demographic characteristics such as older age, lower socio-economic status, living alone, and (co-)morbidity (e.g. diabetes mellitus, renal diseases, hypertension, unstable angina, stroke or transient ischemic attack, cancer, and depression) have been found to be associated with a higher risk of re-infarction and mortality after discharge (4-8). None of these studies have taken into consideration risk factors for re-infarction or mortality in the mid-term i.e. one year after hospital discharge. Moreover, currently there is little evidence related to crucial AMI-related characteristics such as type of coronary revascularisation and infarction. Here, studies are lacking which include a vast range of risk factors and are based on register data, which provide large study populations and guarantee practically no loss to follow-up.

For peer review only

For peer review only - http://bmjopen.bmj.com/site/about/guidelines.xhtml

Additionally, there is a lack of studies elucidating the associations between characteristics of labour market marginalisation and the risk of re-infarction and mortality among AMI patients. In Sweden, more than 30 000 persons experience an AMI each year; of these, about 10 000 are below the age of 65 (9). This burden of disease may result in long-term work disability in the working age population (10, 11). To date, sickness absence (SA) is almost always prescribed as a rehabilitation strategy in healthcare services for patients with AMI (12). Also, permanent work disability, i.e. disability pension (DP), is common in this patient group (10). In a prior study, patterns of SA/DP before AMI provided crucial information for subsequent work disability (13). To the best of our knowledge, this is the first study investigating labour market marginalisation measured in terms of trajectories of SA/DP and unemployment status as risk factors for re-infarction and mortality in patients with AMI.

Aims

The study aimed to investigate to what extent socio-demographic, labour market marginalisation, and medical (including AMI-related factors and co-morbidities) characteristics before/at an AMI were associated with subsequent re-infarction and all-cause mortality during the first year.

For peer review only - http://bmjopen.bmj.com/site/about/guidelines.xhtml

Materials and Methods

Study population

This is a nationwide register-based cohort study and the study population consisted of 16 983 individuals aged 25 to 64 who had a first AMI during 2008-2010. A main diagnosis of AMI was ascertained from the inpatient care register and defined according to the International Classification of Diseases (ICD)-10 code of I21. This means that individuals with a previous main or side diagnosis of AMI in specialised healthcare from 1987 up to the hospital admission date for AMI during 2008-2010 were excluded (n=1914). Altogether, there were 15 069 individuals included in the study.

For peer review only

Registers

National register data was linked to the study population by using the unique personal identity number assigned to all Swedish inhabitants, including information for each individual up to 31st December 2013 from:

1.) Statistics Sweden: sex, age, education, country of birth, type of living area, family situation, length of unemployment, and year of emigration from the Longitudinal integration database for health insurance and labour market studies (LISA);

2.) The Social Insurance Agency: SA/DP (date and grade) from Micro-data for analyses of social insurance (MiDAS);

3.) The National Board of Health and Welfare: date and cause of diagnosis-specific inpatient and specialised outpatient care, and type of infarction and type of coronary revascularisation from the National Patient Register; date of death from the Cause of Death Register (14) and date, type and dose of prescription of dispensed psychiatric medication and antidiabetic medication from the National Prescribed Drug Register.

For peer review only - http://bmjopen.bmj.com/site/about/guidelines.xhtml

Outcome measures

The outcome measures were re-infarction (ICD-codes: I21) which was ascertained from the inpatient care, and all-cause mortality during the first year after AMI.

Risk measures

Socio-demographic characteristics were recorded at the end of the year preceding AMI and comprised: sex, age, education (low educational level (compulsory (≤9 years), high school (10-12 years)), and high educational level (university (>12 years))), country of birth, type of living area , and family situation (Table 1).

Labour market marginalisation characteristics included length of unemployment in the year preceding AMI and the trajectory groups of SA/DP during three years before and up to the AMI diagnosis (Table 1). The trajectory groups of SA/DP were measured using the combined mean number of annual SA and DP net days before the AMI diagnosis. The total number of net days were then transformed to number of months with SA/DP.

Medical characteristics included AMI-related characteristics (type of infarction and type of coronary revascularisation) at inclusion and inpatient and specialised outpatient care due to any main or side diagnosis of somatic and mental co-morbidities and medication which were measured from three years before until the AMI diagnosis. Type of infarction was classified as ST-elevation myocardial infarction (STEMI, ICD-codes: I21.0-I21.3), non-ST-elevation myocardial infarction (NSTEMI, ICD-codes: I21.4), or unspecified (ICD-codes: I21.9). Information on type of coronary revascularisation was categorised as: percutaneous coronary intervention (PCI) (FNG00-FNG05), coronary artery bypass grafting (CABG) (FNA-FNF, FNG30, FNW96), and others (i.e. other treatments/examinations or missing information).

For peer review only - http://bmjopen.bmj.com/site/about/guidelines.xhtml

Somatic co-morbidities were categorised as musculoskeletal diagnoses (ICD-codes: M00-99), renal insufficiency (ICD-codes: N17-N19), stroke (ICD-codes: I60, I61, I63, I64), hypertension (ICD-codes: I10), cancer (ICD-codes: C00-D48), and other somatic disorders (the other ICD-codes except for mental diagnoses). The individuals with any specialised care due to diabetes mellitus or having any prescribed antidiabetic medication were coded according to ICD-codes: E10-E14 and the Anatomic Therapeutic Chemical classification system (ATC) code: A10. Mental co-morbidities were grouped as CMDs (i.e. depressive (ICD-codes: F32-F33), anxiety (ICD-codes: F40-F42) and stress-related disorders (ICD-codes: F43)), and other mental disorders (ICD-codes: F00-F31, F34-F39, and F44-F99). Moreover, prescribed psychiatric medication during the year preceding the AMI diagnosis was included as mental co-morbidities. Psychiatric medication was measured by any antidepressants, anxiolytics and sedatives following the ATC codes, N06A, N05B and N05C, respectively.

Statistical analyses

We used group-based trajectory modelling to estimate groups of SA/DP trajectories during the 3-year period before AMI. This method has been described elsewhere (13, 15). Five groups were selected as the best fitting model for patients with AMI. An annual time-scale was used in the study, where T0 represents the first hospital admission date due to AMI and T-3 represents the 3 years before the first AMI diagnosis (See Fig. 1). The five trajectory groups were named according to the patterns of each group: “Low increasing”, “Low constant”, “Middle increasing”, “High decreasing” and “High constant”.

For peer review only - http://bmjopen.bmj.com/site/about/guidelines.xhtml

Chi-2 tests were used to estimate potential sex differences regarding all the examined characteristics among patients with AMI. Hazard ratios (HR) and 95% confidence intervals (CIs) for re-infarction and all-cause mortality were calculated using Cox regression. The proportional hazards assumption was tested and met. Follow-up time started from the first hospital admission date due to AMI diagnosis until the events (re-infarction or all-cause mortality), emigration to a foreign country, or the end of the first year after AMI, whichever came first. Mean follow-up time for re-infarction and all-cause mortality was 117 days (SD 120) and 177 days (SD 109), respectively. Analyses were adjusted for all risk measures in the multivariate model (mental co-morbidities were not mutually adjusted). Data processing was performed using SAS version 9.4.

Patient and public involvement

There was no patient involvement in this study.

For peer review only - http://bmjopen.bmj.com/site/about/guidelines.xhtml

RESULTS

Table 1 shows descriptive analysis for patients with a first AMI during 2008-2010. Of all, there were 3673 women (24.4%). The majority of the study population was older (56-64 years, 59.3%), born in Sweden (80.2%), belonged to the low increasing SA/DP trajectory group (53.4%) (Fig. 1), were not unemployed before inclusion (91.6%), received PCI at inclusion (68.8%), had other somatic disorders (67.1%), and did not have mental co-morbidities. Re-infarction and all-cause mortality during the first year represented 35.2% and 4.4% of the study population, respectively. Futhermore, sex differences were significant for various factors. For example, with respect to labour market marginalisation characteristics, the “Low increasing” SA/DP group comprised more men (58.8% vs. 36.6%) while the “High constant” SA/DP group was more common for women (22.7% vs. 11.1%). Moreover, more men had a STEMI (36.9% vs. 28.8%) and received a PCI (72.5% vs. 57.2%) compared to women while more women had co-morbidities compared to men.

Re-infarction

In the univariate analyses, higher risks of re-infarction were found in those with lower education and living in small towns/villages. In contrast, those born in non-Nordic European countries, and those living in medium-sized cities had lower risks of subsequent re-infarction during the first year. Moreover, a higher risk of re-infarction was observed among those with STEMI compared to non-STEMI as well as those treated with CABG compared to PCI (HR 2.43; 95% CI 2.14-2.75) (Table 2).

In the final model, lower educational level and living in small towns/villages were associated with a higher risk of re-infarction while living in medium-sized cities, and being single living

For peer review only - http://bmjopen.bmj.com/site/about/guidelines.xhtml

without children at home showed lower risk of re-infarction. With regard to AMI-related characteristics, patients with STEMI and CABG had a higher risk of re-infarction (Table 2).

All-cause mortality

In the multivariable model, we found that older age, lower level of education, being married/single living without children at home, and belonging to the "High constant" SA/DP trajectory group were risk factors for all-cause mortality during the first year after AMI. Those born in non-European countries and those belonging to the "Low constant "and "High decreasing" SA/DP trajectory groups were associated with a lower risk of all-cause mortality. STEMI compared to non-STEMI was associated with a 4-fold higher risk of all-cause mortality following AMI. Moreover, a higher risk of all-cause mortality was found in patients with diabetes mellitus, renal insufficiency, stroke, cancer and other somatic disorders compared to those without such co-morbidities. Other mental disorders besides CMDs and psychiatric medication were significantly associated with subsequent all-cause mortality (Table 3).

For peer review only - http://bmjopen.bmj.com/site/about/guidelines.xhtml

Discussion

Socio-demographic and labour market marginalisation

Socio-demographic and labour market marginalisation were generally more associated with mortality than re-infarction in AMI patients. Further, risk factors in common for both outcomes showed slightly higher risk estimates for mortality. For instance, results showed that a lower education level, which acts as a proxy of lower socioeconomic status, was associated with a less favourable prognosis regarding re-infarction (HR: 1.12) and all-cause mortality (HR: 1.29) during the first year after AMI. Previous studies have shown that patients with a lower educational level generally have a higher risk profile, primarily due to the presence of more risk factors such as smoking or the resistance of quitting smoking after AMI and co-morbidities, leading to a worse health outcome (16, 17). After adjustment for co-morbidities, we found that educational level remained an independent predictor of re-infarction and mortality, Still, one cannot rule out the possibility of unmeasured residual co-morbidities that may be associated with re-infarction and all-cause mortality.

As expected, we observed that higher age was a strong predictor of all-cause mortality after AMI, which is in agreement with other studies (18-20). Similar to individuals with a lower educational level, elderly patients have a greater disease burden and thus are more likely to have a higher risk of mortality. Somewhat unexpectedly, older age was not associated with re-infarction during the first year. The different findings with respect to mortality and re-infarction may be driven by the co-morbidities that were controlled for in the model, which are closely related with AMI and the association between age and all-cause mortality might be caused by other co-morbidites. Interestingly, we found a higher risk of re-infarction for patients who were living in small towns/villages, while a lower risk of re-infarction was

For peer review only

For peer review only - http://bmjopen.bmj.com/site/about/guidelines.xhtml

observed for those living in medium sized cities compared to those living in big cities. This result might indicate diversities in healthcare in relation to different types of living area (21).

Furthermore, AMI patients born in non-European countries had a lower risk of all-cause mortality during the first year than patients born in Sweden. Recent research has shown a lower risk of mortality after AMI among South Asians compared with the host population (22, 23). Our finding may also reflect a "healthy migrant effect", indicating a positive health selection of migrants who are able to overcome the obstacles of migration. Previous studies showed that migrants have revealed a lower risk of morbidity and mortality compared to natives (24, 25).

Compared to AMI patients who were married and living with children at home, those who were married/single and living without children at home had a higher risk of all-cause mortality. Patients who live alone may have poor adherence to medication and follow-up recommendations, which might be associated with an unfavourable outcome. The few studies that have described the association between social support and prognosis in patients with coronary artery disease have had inconsistent definitions of measures of social support, leading to a wide variety of conclusions (26). Therefore, the impact of family situation on re-infarction and all-cause mortality is open to speculation and warrants further investigation.

With regard to labour market marginalisation factors, the "High constant" SA/DP trajectory group was associated with a 2.2-fold higher risk of all-cause mortality, even after controlling for confounders. As this group had around 12 months of SA/DP per annum before AMI, it is likely that this group consisted of a larger proportion of individuals with long-term SA or DP. This group may also have had a history of co-morbidities before AMI, which in turn increases

For peer review only

287 the risk of all-cause mortality. On the other hand, the “Low constant” and “High decreasing”
288 SA/DP trajectory groups showed a lower risk of all-cause mortality after adjusting for co-
289 morbidities. The risk estimates of these two groups were not significant in the univariate
290 model, thereby indicating that the effect of these SA/DP trajectory groups was mainly
291 explained by co-morbidities. Our study is the first to report that SA/DP trajectory groups can
292 be used as risk factors for mortality in patients with AMI. Our findings also revealed that risk
293 estimates of SA/DP trajectory groups were comparable to well-known risk factors such as
294 diabetes mellitus and renal insufficiency. Therefore, more attention in clinical practice in
295 relation to work disabilityfactors in AMI patients is necessary.

296

297 **Medical characteristics**

298 Patients with STEMI had a higher risk of adverse outcomes, particularly for all-cause
299 mortality, while those who underwent CABG had a higher risk of re-infarction than patients
300 with NSTEMI and PCI. Indeed, STEMI is clinically associated with more serious medical
301 conditions than non-STEMI (27). Several studies have suggested that STEMI patients exhibit
302 an adverse prognosis in clinical settings due to related co-morbidities or the
303 pathophysiological nature of STEMI (28, 29). With respect to coronary revascularisation, the
304 outcome of PCI and CABG on AMI patients is inconsistent across studies. Although some
305 studies have found similar morbidities and mortalities for PCI and CABG in AMI patients,
306 others have shown that patients treated with PCI rather than CABG had fewer complications
307 and a lower risk of mortality, particularly in the short-term (30, 31). It might also be due to the
308 fact that patients with severe coronary artery disease are often treated with CABG and CABG
309 is a more invasive method compared to PCI. On the other hand, some studies have also shown
310 satisfying outcomes for those patients who were treated with CABG at 1 year after AMI (32).
311 Therefore, the role of coronary revascularisation needs to be further investigated.

For peer review only - http://bmjopen.bmj.com/site/about/guidelines.xhtml

We also found several co-morbidities such as diabetes mellitus, renal insufficiency, stroke, and cancer that were associated with a higher risk of all-cause mortality after AMI, which is in agreement with previous studies (33-35). Because patients with a greater disease burden are more likely to experience higher mortality rates than their healthier counterparts, future studies adressing surveillance and treatment approaches for patients presenting with multiple co-morbidities are necessary. In contrast, diabetes mellitus was associated with a lower risk of re-infarction.

In addition, a higher risk of all-cause mortality was observed among patients with mental co-morbidities. Both biological and behavioural mechanisms have been suggested to explain the association between mental disorders and cardiovascular disease. Patients with mental disorders have been reported to have several cardiac symptoms (36, 37). Further, they tend to have poorer diets, reduced medication adherence, and more stress (38). Overall, mental disorders reduce the success of interventions targeting cardiovascular risk factor modification, leading to higher healthcare costs, poorer health outcomes, and increased mortality rates.

Strengths and limitations

The strengths of this study include the use of a population-based cohort design, which offers satisfactory statistical power for the analyses. The use of high quality national register data also minimises the risk of recall bias regarding exposure and outcome (39). The high coverage of the register data also enabled us to identify all AMI patients from inpatient care with subsequent re-infarction and mortality. We included only AMI patients who were treated in inpatient care with more severe cardiac disease. This might explain the high incidence of re-infarction during the first year of the study. We also used an advanced method covering the

For peer review only

For peer review only - http://bmjopen.bmj.com/site/about/guidelines.xhtml

inherent heterogeneity, group-based trajectory modelling, to investigate work disability patterns in the study. Moreover, we were able to examine a wide range of risk factors as well as adjust for relevant confounders. Still, there might be other factors than those studied here that are associated with re-infarction and mortality. Our registers did not include information of compliance to prescribed medication such as dual-antiplatelet therapy, smoking habits before and after AMI, rehabilitation measures and life-style changes.

Limitations of the study and considerations when interpreting our findings are acknowledged. In this study, we only included co-morbidities recorded in inpatient and specialised outpatient care, but not those from primary care due to lack of data availability. While we adjusted for potential confounders that were particularly relevant for AMI, we acknowledge that there may be a wider range of co-morbidities that we were unable to control for. Mental co-morbidities were measured by including prescribed psychiatric medication data. For somatic co-morbidities, we did not include an equivalent measure except for diabetes mellitus as there was no available information in the register data. With regard to sickness absence, we did not have information on sick-leave spells that were less than 14 days among employed individuals. Thus, the number of SA days contributing to the combined number of SA/DP days might be underestimated.

For peer review only

Conclusions

Several socio-demographic and co-morbidity risk factors were generally associated more strongly with mortality than re-infarction in AMI patients, including lower educational level, older age, immigration status, somatic and mental co-morbidities. Previous long-term work disability and infarction type showed a higher risk for all-cause mortality after AMI during the first year.

For peer review only - http://bmjopen.bmj.com/site/about/guidelines.xhtml

Author Contributions

EM, MV and MW conceived and designed the study. MW and EM were involved in the statistical analysis and drafted the manuscript. All authors gave input to the drafts and approved the final manuscript.

Acknowledgements

None.

Funding

This study was supported by the Swedish Research Council, grant nr 2015-02292.

Declaration of conflicting interests

None.

Ethics approval

The study has been evaluated and approved by the Regional Ethical Review Board of Karolinska Institutet, Stockholm, Sweden (2007/762–31). The ethical review board approved the study and waived the requirement that informed consent of research subjects should be obtained.

Data sharing statement

The data that support the findings of this study are available from Statistics Sweden and The Swedish National Board of Health and Welfare, but restrictions apply to the availability of

For peer review only - http://bmjopen.bmj.com/site/about/guidelines.xhtml

385 these data, which were used with ethical permission for the current study and therefore are not

386 publicly available.

For peer review only - http://bmjopen.bmj.com/site/about/guidelines.xhtml

References

1. Fiscella K, Meldrum S, Barnett S. Hospital discharge against advice after myocardial infarction: deaths and readmissions. The American journal of medicine. 2007;120(12):1047-53.
2. Mohr FW, Morice MC, Kappetein AP, Feldman TE, Stahle E, Colombo A, et al. Coronary artery bypass graft surgery versus percutaneous coronary intervention in patients with three-vessel disease and left main coronary disease: 5-year follow-up of the randomised, clinical SYNTAX trial. Lancet. 2013;381(9867):629-38.
3. Chung SC, Gedeborg R, Nicholas O, James S, Jeppsson A, Wolfe C, et al. Acute myocardial infarction: a comparison of short-term survival in national outcome registries in Sweden and the UK. Lancet (London, England). 2014;383(9925):1305-12.
4. Dunlay SM, Weston SA, Killian JM, Bell MR, Jaffe AS, Roger VL. Thirty-day rehospitalizations after acute myocardial infarction: a cohort study. Annals of internal medicine. 2012;157(1):11-8.
5. Khawaja FJ, Shah ND, Lennon RJ, Slusser JP, Alkatib AA, Rihal CS, et al. Factors associated with 30-day readmission rates after percutaneous coronary intervention. Archives of internal medicine. 2012;172(2):112-7.
6. Kamalesh M, Subramanian U, Ariana A, Sawada S, Tierney W. Similar decline in post-myocardial infarction mortality among subjects with and without diabetes. The American journal of the medical sciences. 2005;329(5):228-33.
7. Brown JR, Conley SM, Niles NW, 2nd. Predicting readmission or death after acute ST-elevation myocardial infarction. Clinical cardiology. 2013;36(10):570-5.
8. Agrawal Y, Panaich S, Aggarwal S, Saltiel F, Kalavakunta JK, Gupta V. Demographic parameters related to 30-day readmission of patients with acute myocardial infarction: Analysis of 2,371,867 hospitalizations. International journal of cardiology. 2016;214:408-9.
9. Annual report of SWEDEHEART 2010 (Årsrapport SWEDEHEART 2010. RIKS-HIA, SEPHIA, SCAAR, Hjärtkirurgi och TAVI. In Swedish) [Available from: http://www.ucr.uu.se/swedeheart/dokument-sh/arsrapporter/aeldre-arsrapporter-older-reports/arsrapporter-2007-2010.
10. Zetterstrom K, Vaez M, Alexanderson K, Ivert T, Pehrsson K, Hammar N, et al. Disability pension after coronary revascularization: a prospective nationwide register-based Swedish cohort study. Eur J Prev Cardiol. 2015;22(3):304-11.
11. Smedegaard L, Nume AK, Charlot M, Kragholm K, Gislason G, Hansen PR. Return to Work and Risk of Subsequent Detachment From Employment After Myocardial Infarction: Insights From Danish Nationwide Registries. Journal of the American Heart Association. 2017;6(10).
12. National Board of Health and Welfare (NBHW, Socialstyrelsen): Basis for descision in insurance medicine (försäkringsmedicinskt beslutstöd) 2015 [Available from: http://www.socialstyrelsen.se/riktlinjer/forsakringsmedicinsktbeslutsstod.
13. Wang M, Vaez M, Dorner TE, Tiihonen J, Voss M, Ivert T, et al. Trajectories and characteristics of work disability before and after acute myocardial infarction. Heart. 2017.
14. Socialstyrelsen. Dödsorsaksregistret, Patientregistret. [Cause of Death register, National Patient Register; online resource, in Swedish]. . Stockholm: National Board of Health and Welfare; 2009.
15. Jones B, Nagin D, Roeder K. A SAS Procedure Based on Mixture Models for Estimating Developmental Trajectories. Sociological Methods & Research 2001;29 (3):374-93.
16. Mehta RH, O'Shea JC, Stebbins AL, Granger CB, Armstrong PW, White HD, et al. Association of mortality with years of education in patients with ST-segment elevation

For peer review only - http://bmjopen.bmj.com/site/about/guidelines.xhtml

myocardial infarction treated with fibrinolysis. Journal of the American College of Cardiology. 2011;57(2):138-46.
17. Lee KL, Woodlief LH, Topol EJ, Weaver WD, Betriu A, Col J, et al. Predictors of 30-day mortality in the era of reperfusion for acute myocardial infarction. Results from an international trial of 41,021 patients. GUSTO-I Investigators. Circulation. 1995;91(6):1659-68.
18. Waldecker B, Waas W, Haberbosch W, Voss R, Heizmann H, Tillmanns H. Long-term follow-up after direct percutaneous transluminal coronary angioplasty for acute myocardial infarction. Journal of the American College of Cardiology. 1998;32(5):1320-5.
19. Califf RM, Pieper KS, Lee KL, Van De Werf F, Simes RJ, Armstrong PW, et al. Prediction of 1-year survival after thrombolysis for acute myocardial infarction in the global utilization of streptokinase and TPA for occluded coronary arteries trial. Circulation. 2000;101(19):2231-8.
20. Lee CH, van Domburg RT, Hoye A, Lemos PA, Tanabe K, Smits PC, et al. Predictors of survival after contemporary percutaneous coronary revascularization for acute myocardial infarction in the real world. The Journal of invasive cardiology. 2004;16(11):627-31.
21. Caldwell JT, Ford CL, Wallace SP, Wang MC, Takahashi LM. Intersection of Living in a Rural Versus Urban Area and Race/Ethnicity in Explaining Access to Health Care in the United States. American journal of public health. 2016;106(8):1463-9.
22. Bansal N, Fischbacher CM, Bhopal RS, Brown H, Steiner MF, Capewell S. Myocardial infarction incidence and survival by ethnic group: Scottish Health and Ethnicity Linkage retrospective cohort study. BMJ open. 2013;3(9):e003415.
23. Zaman MJ, Philipson P, Chen R, Farag A, Shipley M, Marmot MG, et al. South Asians and coronary disease: is there discordance between effects on incidence and prognosis? Heart (British Cardiac Society). 2013;99(10):729-36.
24. Di Thiene D, Alexanderson K, Tinghog P, La Torre G, Mittendorfer-Rutz E. Suicide among first-generation and second-generation immigrants in Sweden: association with labour market marginalisation and morbidity. J Epidemiol Community Health. 2015;69(5):467-73.
25. Guillot M, Khlat M, Elo I, Solignac M, Wallace M. Understanding age variations in the migrant mortality advantage: An international comparative perspective. PloS one. 2018;13(6):e0199669.
26. Case RB, Moss AJ, Case N, McDermott M, Eberly S. Living alone after myocardial infarction. Impact on prognosis. Jama. 1992;267(4):515-9.
27. Anderson JL, Morrow DA. Acute Myocardial Infarction. The New England journal of medicine. 2017;376(21):2053-64.
28. Goldberg A, Hammerman H, Petcherski S, Zdorovyak A, Yalonetsky S, Kapeliovich M, et al. Inhospital and 1-year mortality of patients who develop worsening renal function following acute ST-elevation myocardial infarction. American heart journal. 2005;150(2):330-7.
29. Masci PG, Ganame J, Francone M, Desmet W, Lorenzoni V, Iacucci I, et al. Relationship between location and size of myocardial infarction and their reciprocal influences on post-infarction left ventricular remodelling. European heart journal. 2011;32(13):1640-8.
30. Stone GW, White HD, Ohman EM, Bertrand ME, Lincoff AM, McLaurin BT, et al. Bivalirudin in patients with acute coronary syndromes undergoing percutaneous coronary intervention: a subgroup analysis from the Acute Catheterization and Urgent Intervention Triage strategy (ACUITY) trial. Lancet (London, England). 2007;369(9565):907-19.
31. Hochholzer W, Buettner HJ, Trenk D, Breidthardt T, Noveanu M, Laule K, et al. Percutaneous coronary intervention versus coronary artery bypass grafting as primary revascularization in patients with acute coronary syndrome. The American journal of cardiology. 2008;102(2):173-9.

For peer review only - http://bmjopen.bmj.com/site/about/guidelines.xhtml

485 32. Serruys PW, Morice MC, Kappetein AP, Colombo A, Holmes DR, Mack MJ, et al.
486 Percutaneous coronary intervention versus coronary-artery bypass grafting for severe
487 coronary artery disease. The New England journal of medicine. 2009;360(10):961-72.
488 33. David RB, Almeida ED, Cruz LV, Sebben JC, Feijo IP, Schmidt KE, et al. Diabetes
489 mellitus and glucose as predictors of mortality in primary coronary percutaneous intervention.
490 Arquivos brasileiros de cardiologia. 2014;103(4):323-30.
491 34. Lazaros G, Tsiachris D, Tousoulis D, Patialiakas A, Dimitriadis K, Roussos D, et al. In-
492 hospital worsening renal function is an independent predictor of one-year mortality in patients
493 with acute myocardial infarction. International journal of cardiology. 2012;155(1):97-101.
494 35. Rasoul S, Ottervanger JP, de Boer MJ, Dambrink JH, Hoorntje JC, Marcel Gosselink AT,
495 et al. Predictors of 30-day and 1-year mortality after primary percutaneous coronary
496 intervention for ST-elevation myocardial infarction. Coronary artery disease. 2009;20(6):415-
497 21.
498 36. Sherwood A, Hinderliter AL, Watkins LL, Waugh RA, Blumenthal JA. Impaired
499 endothelial function in coronary heart disease patients with depressive symptomatology.
500 Journal of the American College of Cardiology. 2005;46(4):656-9.
501 37. Empana JP, Sykes DH, Luc G, Juhan-Vague I, Arveiler D, Ferrieres J, et al. Contributions
502 of depressive mood and circulating inflammatory markers to coronary heart disease in healthy
503 European men: the Prospective Epidemiological Study of Myocardial Infarction (PRIME).
504 Circulation. 2005;111(18):2299-305.
505 38. Lett HS, Blumenthal JA, Babyak MA, Sherwood A, Strauman T, Robins C, et al.
506 Depression as a risk factor for coronary artery disease: evidence, mechanisms, and treatment.
507 Psychosomatic medicine. 2004;66(3):305-15.
508 39. Ludvigsson JF, Andersson E, Ekbom A, Feychting M, Kim JL, Reuterwall C, et al.
509 External review and validation of the Swedish national inpatient register. BMC public health.
510 2011;11:450.

511

512

For peer review only - http://bmjopen.bmj.com/site/about/guidelines.xhtml

Risk factors for re-infarction and mortality after acute myocardial infarction

Table 1: Descriptive statistics for all women (n=3673) and men (n=11 396) aged between 25 and 64 years with a diagnosis of acute myocardial infarction (AMI) from inpatient care in 2008-2010 in Sweden (N=15 069)

Characteristics of patients with AMI	All		Women		Men		Chi^2 (p-value)
	n 15 069	% 100	n 3673	% 24.4	n 11 396	% 75.6	
**Socio-demographic characteristics**[a]							
_**Age**_*							
25-45	1401	9.3	335	9.1	1066	9.4	15.1 (<0.001)
46-55	4739	31.5	1065	29.0	3674	32.2	
56-64	8929	59.3	2273	61.9	6656	58.4	
_**Education (years)**_*[b]							
Compulsory (≤9)	4474	29.7	1040	28.3	3434	30.1	9.9 (<0.01)
High school (10–12)	7435	49.3	1895	51.6	5540	48.6	
University (>12)	3160	21.0	738	20.1	2422	21.3	
_**Country of birth**_*[c]							
Sweden	12 085	80.2	2991	81.4	9094	79.8	86.4 (<0.001)
Other Nordic countries	860	5.7	282	7.7	578	5.1	
Europe (except Nordic countries)	440	2.9	116	3.2	324	2.8	
Non-European countries	1684	11.2	284	7.7	1400	12.3	
_**Type of living area**_*[d]							
Big cities	4566	30.3	1052	28.6	3514	30.8	6.5 (<0.05)
Medium sized cities	5347	35.5	1344	36.6	4003	35.1	
Small towns/villages	5156	34.2	1277	34.8	3879	34.0	
_**Family situation**_*[e]							
Married[f] living without children	4880	32.4	1342	36.5	3538	31.1	235.8 (<0.001)
Married[f] living with children	4000	26.5	728	19.8	3272	28.7	
Single[g] living without children	5386	35.7	1271	34.6	4115	36.1	
Single[g] living with children	803	5.3	332	9.0	471	4.1	
**Labour market marginalisation characteristics**							
_**Trajectory groups of SA/DP**_* _**from three years before up to inclusion**_							
Low increasing	8048	53.4	1345	36.6	6703	58.8	705.4 (<0.001)
Low constant	2714	18.0	709	19.3	2005	17.6	
Middle increasing	1420	9.4	455	12.4	965	8.5	
High decreasing	794	5.3	331	9.0	463	4.1	
High constant	2093	13.9	833	22.7	1260	11.1	
_**Unemployment**_* _**in the year before inclusion**_							
No unemployment	13 799	91.6	3420	93.1	10 379	91.1	15.0 (<0.001)
1-180 days	852	5.7	171	4.7	681	6.0	
>180 days	418	2.8	82	2.2	336	3.0	
**AMI-related characteristics**							
_**Type of infarction**_*[h] _**at inclusion**_							
STEMI[i]	5260	34.9	1058	28.8	4202	36.9	84.7 (<0.001)
Non-STEMI[j]	6704	44.5	1832	49.9	4872	42.8	

For peer review only

For peer review only - http://bmjopen.bmj.com/site/about/guidelines.xhtml

Unspecified	3105	20.6	783	21.3	2322	20.4	
Coronary revascularisation characteristics[*k] at inclusion							
Percutaneous coronary intervention	10 364	68.8	2100	57.2	8264	72.5	353.9 (<0.001)
Coronary artery bypass grafting	336	2.2	59	1.6	277	2.4	
Others	4369	29.0	1514	41.2	2855	25.1	
**Co-morbidity characteristics[h]**							
**Somatic co-morbidities[l] from three years before up to inclusion**							
Musculoskeletal disorders*	2299	15.3	741	20.2	1558	13.7	90.9 (<0.001)
Diabetes mellitus[*m]	2529	16.8	675	18.4	1854	16.3	8.8 (<0.01)
Renal insufficiency	248	1.7	70	1.9	178	1.6	2.0 (0.15)
Hypertension*	5110	33.9	1365	37.2	3745	32.9	22.9 (<0.001)
Stroke	199	1.3	54	1.5	145	1.3	0.8 (0.36)
Cancer*	933	6.2	303	8.3	630	5.5	35.4 (<0.001)
Other somatic disorders*	10 107	67.1	2722	74.1	7385	64.8	108.9 (<0.001)
**Mental co-morbidities**							
Common mental disorders[*l] from three years before up to inclusion	791	5.3	287	7.8	504	4.4	64.2 (<0.001)
Other mental disorders[l] from three years before up to inclusion	1331	8.8	328	8.9	1003	8.8	0.1 (0.81)
Psychiatric medication[*n] in the year before inclusion	3231	21.4	1299	35.4	1932	16.7	559.1 (<0.001)
**Re-infarction and all-cause mortality during first year after AMI**							
Re-infarction	5310	35.2	1276	34.7	4034	35.4	0.5 (0.47)
All-cause mortality*	666	4.4	191	5.2	475	4.2	7.0 (<0.01)

* Significant sex differences
[a] Measured on 31st December of the year preceding acute myocardial infarction
[b] Missing data is considered compulsory education
[c] Missing data is considered Non-European countries
[d] Type of living area: big cities (Stockholm, Gothenburg and Malmö); medium sized cities (cities with more than 90 000 inhabitants within 30 km distance from the centre of the city); small cities/villages/rural
[e] Missing data is considered single living without children
[f] Married includes all living with partner; cohabitant
[g] Single includes divorced, separated, or widowed
[h] See method section for the International Classification of Diseases version 10 (ICD-10) codes or the Anatomic Therapeutic Chemical classification system (ATC) codes
[i] ST-elevation myocardial infarction
[j] Non-ST-elevation myocardial infarction
[k] See method section for the Classification of Surgical Procedures
[l] Measured by main or side diagnosis in inpatient or specialised outpatient care
[m] Additionally measured by prescribed antidiabetic medication
[n] Measured by antidepressants, anxiolytics and sedatives

For peer review only

For peer review only - http://bmjopen.bmj.com/site/about/guidelines.xhtml

Risk factors for re-infarction and mortality after acute myocardial infarction

Table 2. Crude and adjusted hazard ratios (HR) and 95% Confidence Interval (CI) for re-infarction in individuals with a diagnosis of acute myocardial infarction (AMI) from inpatient care in 2008-2010 in Sweden (N=15 069) during the first year after AMI

Characteristics of patients with AMI	**Re-infarction**	Crude model	Model 1[a]	Model 2[b]	Model 3[c]
	n (%)	HR (95% CI)			
**Socio-demographic characteristics[d]**					
Sex					
Men	4034 (35.4)	1	1	1	1
Women	1276 (34.7)	0.97 (0.91-1.03)	0.97 (0.91-1.04)	0.98 (0.91-1.04)	1.03 (0.97-1.11)
Age					
25-45	482 (34.4)	1	1	1	1
46-55	1679 (35.4)	1.03 (0.93-1.14)	1.02 (0.92-1.13)	1.02 (0.92-1.13)	1.01 (0.91-1.12)
56-64	3149 (35.3)	1.01 (0.92-1.12)	0.98 (0.89-1.09)	0.99 (0.90-1.10)	0.99 (0.90-1.10)
Education (years)[e]					
Compulsory (≤9)	1630 (36.4)	1.18 (1.09-1.28)	1.13 (1.05-1.23)	1.13 (1.04-1.23)	1.12 (1.04-1.22)
High school (10–12)	2672 (35.9)	1.16 (1.08-1.25)	1.11 (1.03-1.19)	1.11 (1.03-1.19)	1.10 (1.02-1.18)
University (>12)	1008 (31.9)	1	1	1	1
Country of birth[f]					
Sweden	4367 (36.1)	1	1	1	1
Other Nordic countries	309 (35.9)	1.00 (0.89-1.12)	1.01 (0.90-1.13)	1.01 (0.90-1.13)	1.02 (0.91-1.14)
Europe (except Nordic countries)	124 (28.2)	0.74 (0.62-0.88)	0.83 (0.69-0.99)	0.83 (0.69-0.99)	0.83 (0.70-1.00)
Non-European countries	510 (30.3)	0.80 (0.73-0.87)	0.91 (0.82-1.00)	0.91 (0.82-1.00)	0.91 (0.82-1.00)
Type of living area[g]					
Big cities	1368 (30.0)	1	1	1	1
Medium sized cities	1428 (26.7)	0.87 (0.80-0.93)	0.84 (0.78-0.91)	0.84 (0.78-0.91)	0.84 (0.78-0.91)
Small towns/villages	2514 (48.8)	1.88 (1.76-2.01)	1.81 (1.69-1.94)	1.81 (1.69-1.94)	1.83 (1.71-1.96)
Family situation[h]					
Married[i] living without children	1782 (36.5)	1.01 (0.95-1.09)	0.97 (0.90-1.05)	0.97 (0.90-1.05)	0.97 (0.90-1.05)
Married[i] living with children	1439 (36.0)	1	1	1	1
Single[j] living without children	1812 (33.6)	0.92 (0.86-0.98)	0.89 (0.83-0.96)	0.89 (0.83-0.96)	0.89 (0.83-0.96)
Single[j] living with children	277 (34.5)	0.94 (0.83-1.07)	0.96 (0.84-1.09)	0.96 (0.84-1.09)	0.96 (0.84-1.10)

For peer review only - http://bmjopen.bmj.com/site/about/guidelines.xhtml

Risk factors for re-infarction and mortality after acute myocardial infarction

**Labour market marginalisation characteristics**					
Trajectory groups of SA/DP from three years before up to inclusion					
Low increasing	2827 (35.1)	1	1	1	1
Low constant	951 (35.0)	0.99 (0.92-1.06)	0.96 (0.89-1.04)	0.96 (0.89-1.04)	0.97 (0.90-1.04)
Middle increasing	501 (35.3)	0.97 (0.88-1.07)	0.95 (0.87-1.06)	0.96 (0.87-1.05)	0.99 (0.89-1.09)
High decreasing	295 (37.2)	1.03 (0.91-1.16)	1.02 (0.90-1.16)	1.02 (0.90-1.16)	1.08 (0.95-1.23)
High constant	736 (35.2)	0.99 (0.91-1.07)	1.00 (0.92-1.09)	1.00 (0.91-1.09)	1.06 (0.97-1.17)
Unemployment in the year before inclusion					
No unemployment	4850 (35.2)	1	1	1	1
1-180 days	310 (36.4)	1.04 (0.93-1.17)	1.07 (0.95-1.20)	1.07 (0.95-1.20)	1.06 (0.94-1.19)
>180 days	150 (35.9)	1.04 (0.89-1.23)	1.08 (0.92-1.27)	1.08 (0.92-1.27)	1.07 (0.91-1.26)
**AMI-related characteristics**					
Type of infarction[k] at inclusion					
STEMI[l]	1945 (37.0)	1.18 (1.11-1.26)	1.19 (1.12-1.27)	1.19 (1.12-1.27)	1.14 (1.07-1.21)
Non-STEMI[m]	2311 (34.5)	1	1	1	1
Unspecified	1054 (34.0)	1.02 (0.95-1.09)	1.02 (0.95-1.10)	1.02 (0.95-1.10)	1.05 (0.98-1.13)
Coronary revascularisation characteristics[n] at inclusion					
Percutaneous coronary intervention	3772 (36.4)	1	1	1	1
Coronary artery bypass grafting	267 (79.5)	2.43 (2.14-2.75)	2.31 (2.04-2.62)	2.32 (2.05-2.63)	2.41 (2.13-2.74)
Others	1271 (29.1)	0.74 (0.70-0.79)	0.71 (0.67-0.76)	0.71 (0.67-0.76)	0.74 (0.69-0.79)
**Co-morbidity characteristics[k]**					
**Somatic co-morbidities[o] from three years before up to inclusion**					
Musculoskeletal disorders	835 (36.3)	1.04 (0.96-1.12)	1.04 (0.96-1.12)	1.04 (0.96-1.12)	1.05 (0.98-1.14)
Diabetes mellitus[p]	827 (32.7)	0.87 (0.81-0.94)	0.87 (0.81-0.94)	0.87 (0.81-0.94)	0.91 (0.84-0.98)
Renal insufficiency	73 (29.4)	0.77 (0.61-0.97)	0.74 (0.58-0.93)	0.74 (0.59-0.94)	0.84 (0.66-1.06)

For peer review only - http://bmjopen.bmj.com/site/about/guidelines.xhtml

Risk factors for re-infarction and mortality after acute myocardial infarction

Hypertension	1741 (34.1)	0.92 (0.87-0.97)	0.92 (0.87-0.98)	0.93 (0.87-0.98)	0.95 (0.89-1.00)
Stroke	59 (29.7)	0.80 (0.62-1.03)	0.76 (0.59-0.99)	0.77 (0.59-0.99)	0.81 (0.63-1.05)
Cancer	300 (32.2)	0.88 (0.79-0.99)	0.89 (0.79-1.00)	0.90 (0.80-1.01)	0.94 (0.83-1.06)
Other somatic disorders	3507 (34.7)	0.92 (0.87-0.98)	0.92 (0.87-0.97)	0.92 (0.87-0.97)	0.95 (0.90-1.01)
**Mental co-morbidities**					
Common mental disorders[o] from three years before up to inclusion	300 (37.9)	1.08 (0.96-1.21)	1.12 (1.00-1.27)	–	1.13 (1.00-1.28)
Other mental disorders[o] from three years before up to inclusion	474 (35.6)	1.02 (0.93-1.12)	1.06 (0.96-1.17)	–	1.05 (0.96-1.16)
Psychiatric medication[q] in the year before inclusion	1101 (34.1)	0.94 (0.88-1.00)	0.96 (0.90-1.04)	–	1.00 (0.93-1.07)

[a] Adjusted for sex, age, educational level, country of birth, type of living area, family situation, trajectory groups of SA/DP and previous unemployment

[b] Adjusted for sex, age, educational level, country of birth, type of living area, family situation, trajectory groups of SA/DP and previous unemployment, inpatient and specialised outpatient care due to common mental disorders and other mental disorders, and psychiatric medications; Mental co-morbidities were not mutually controlled

[c] Adjusted for sex, age, educational level, country of birth, type of living area, family situation, trajectory groups of SA/DP and previous unemployment, inpatient and specialised outpatient care due to common mental disorders and other mental disorders, and psychiatric medications, type of infarction, type of coronary revascularisation, musculoskeletal disorders, diabetes mellitus, renal insufficiency, hypertension, stroke, cancer and other somatic disorders; Mental co-morbidities were not mutually controlled

[d] Measured on 31st December of the year preceding acute myocardial infarction

[e] Missing data is considered compulsory education

[f] Missing data is considered Non-European countries

[g] Type of living area: big cities (Stockholm, Gothenburg and Malmö); medium sized cities (cities with more than 90 000 inhabitants within 30 km distance from the centre of the city); small cities/villages/rural

[h] Missing data is considered single living without children

[i] Married includes all living with partner; cohabitant

[j] Single includes divorced, separated, or widowed

[k] See method section for the International Classification of Diseases version 10 (ICD-10) codes or the Anatomic Therapeutic Chemical classification system (ATC) codes

[l] ST-elevation myocardial infarction

[m] Non-ST-elevation myocardial infarction

[n] See method section for the Classification of Surgical Procedures

[o] Measured by main or side diagnosis in inpatient or specialised outpatient care

[p] Additionally measured by prescribed antidiabetic medication

[q] Measured by antidepressants, anxiolytics and sedatives

For peer review only

For peer review only - http://bmjopen.bmj.com/site/about/guidelines.xhtml

Risk factors for re-infarction and mortality after acute myocardial infarction

Table 3. Crude and adjusted hazard ratios (HR) and 95% Confidence Interval (CI) for all-cause mortality in individuals with a diagnosis of acute myocardial infarction (AMI) from inpatient care in 2008-2010 in Sweden (N=15 069) during the first year after AMI

Characteristics of patients with AMI	**Mortality**	Crude model	Model 1[a]	Model 2[b]	Model 3[c]
	n (%)	HR (95% CI)			
**Socio-demographic characteristics[d]**					
Sex					
Men	475 (4.2)	1	1	1	1
Women	191 (5.2)	1.25 (1.05-1.47)	0.95 (0.80-1.14)	0.92 (0.78-1.10)	0.94 (0.78-1.12)
Age					
25-45	35 (2.5)	1	1	1	1
46-55	137 (2.9)	1.15 (0.80-1.67)	0.98 (0.67-1.42)	0.97 (0.66-1.40)	1.04 (0.71-1.51)
56-64	494 (5.5)	2.23 (1.58-3.14)	1.76 (1.23-2.50)	1.74 (1.22-2.48)	1.82 (1.27-2.60)
Education (years)[e]					
Compulsory (≤9)	255 (5.7)	1.72 (1.37-2.15)	1.28 (1.02-1.62)	1.29 (1.02-1.63)	1.29 (1.02-1.62)
High school (10–12)	305 (4.1)	1.24 (0.99-1.54)	1.06 (0.85-1.33)	1.06 (0.84-1.32)	1.05 (0.84-1.31)
University (>12)	106 (3.4)	1	1	1	1
Country of birth[f]					
Sweden	550 (4.6)	1	1	1	1
Other Nordic countries	52 (6.1)	1.34 (1.01-1.79)	1.02 (0.77-1.36)	1.03 (0.78-1.38)	1.22 (0.91-1.62)
Europe (except Nordic countries)	16 (3.6)	0.78 (0.48-1.28)	0.70 (0.42-1.15)	0.73 (0.44-1.20)	0.80 (0.48-1.32)
Non-European countries	48 (2.9)	0.62 (0.46-0.83)	0.58 (0.43-0.80)	0.59 (0.44-0.81)	0.62 (0.45-0.84)
Type of living area[g]					
Big cities	214 (4.7)	1	1	1	1
Medium sized cities	224 (4.2)	0.90 (0.74-1.08)	0.84 (0.69-1.02)	0.84 (0.69-1.01)	0.88 (0.72-1.06)
Small towns/villages	228 (4.4)	0.95 (0.79-1.14)	0.84 (0.69-1.01)	0.85 (0.70-1.03)	0.86 (0.71-1.05)
Family situation[h]					
Married[i] living without children	208 (4.3)	1.83 (1.43-2.33)	1.31 (1.02-1.70)	1.30 (1.01-1.68)	1.36 (1.05-1.76)
Married[i] living with children	94 (2.4)	1	1	1	1
Single[j] living without children	334 (6.2)	2.69 (2.14-3.38)	1.75 (1.38-2.22)	1.69 (1.33-2.14)	1.73 (1.36-2.20)
Single[j] living with children	30 (3.7)	1.59 (1.06-2.40)	1.28 (0.84-1.94)	1.25 (0.83-1.90)	1.28 (0.84-1.94)

For peer review only - http://bmjopen.bmj.com/site/about/guidelines.xhtml

Risk factors for re-infarction and mortality after acute myocardial infarction

**Labour market marginalisation characteristics**					
Trajectory groups of SA/DP from three years before up to inclusion					
Low increasing	243 (3.0)	1	1	1	1
Low constant	69 (2.5)	0.84 (0.64-1.10)	0.82 (0.63-1.07)	0.79 (0.60-1.03)	0.68 (0.52-0.89)
Middle increasing	71 (5.0)	1.66 (1.27-2.16)	1.60 (1.22-2.09)	1.47 (1.12-1.93)	1.02 (0.78-1.35)
High decreasing	18 (2.3)	0.77 (0.48-1.24)	0.59 (0.36-0.95)	0.52 (0.32-0.85)	0.33 (0.20-0.54)
High constant	265 (12.7)	4.32 (3.63-5.14)	3.94 (3.26-4.76)	3.45 (2.81-4.23)	2.16 (1.75-2.70)
Unemployment in the year before inclusion					
No unemployment	622 (4.5)	1	1	1	1
1-180 days	27 (3.2)	0.69 (0.47-1.02)	1.07 (0.72-1.59)	1.05 (0.71-1.56)	1.04 (0.70-1.55)
>180 days	17 (4.1)	0.90 (0.56-1.46)	1.31 (0.81-2.14)	1.31 (0.81-2.14)	1.28 (0.79-2.09)
**AMI-related characteristics**					
Type of infarction[k] at inclusion					
STEMI[l]	267 (5.1)	2.28 (1.87-2.78)	2.45 (2.01-2.99)	2.48 (2.03-3.03)	3.80 (3.08-4.68)
Non-STEMI[m]	153 (2.3)	1	1	1	1
Unspecified	246 (7.9)	3.60 (2.94-4.41)	3.45 (2.82-4.23)	3.45 (2.82-4.22)	2.97 (2.42-3.65)
Coronary revascularisation characteristics[n] at inclusion					
Percutaneous coronary intervention	257 (2.5)	1	1	1	1
Coronary artery bypass grafting	11 (3.3)	1.35 (0.74-2.47)	1.26 (0.69-2.31)	1.27 (0.70-2.33)	1.65 (0.90-3.02)
Others	398 (9.1)	3.78 (3.23-4.42)	3.30 (2.81-3.87)	3.25 (2.76-3.81)	3.60 (3.03-4.28)
**Co-morbidity characteristics[k]**					
**Somatic co-morbidities[o] from three years before up to inclusion**					
Musculoskeletal disorders	112 (4.9)	1.12 (0.92-1.38)	0.90 (0.73-1.11)	0.91 (0.74-1.12)	1.00 (0.81-1.24)
Diabetes mellitus[p]	199 (7.9)	2.14 (1.81-2.53)	1.73 (1.46-2.05)	1.74 (1.47-2.06)	1.70 (1.42-2.03)
Renal insufficiency	60 (24.2)	6.36 (4.88-8.30)	4.34 (3.31-5.71)	4.29 (3.26-5.64)	2.59 (1.95-3.45)
Hypertension	198 (3.9)	0.82 (0.69-0.97)	0.74 (0.62-0.87)	0.74 (0.62-0.87)	0.68 (0.57-0.81)

For peer review only

For peer review only - http://bmjopen.bmj.com/site/about/guidelines.xhtml

Risk factors for re-infarction and mortality after acute myocardial infarction

Stroke	25 (12.6)	3.11 (2.09-4.64)	1.96 (1.31-2.94)	1.99 (1.33-2.98)	1.63 (1.09-2.45)
Cancer	102 (10.9)	2.79 (2.26-3.44)	2.46 (1.99-3.05)	2.45 (1.98-3.04)	2.22 (1.78-2.75)
Other somatic disorders	530 (5.2)	1.93 (1.60-2.33)	1.62 (1.34-1.96)	1.59 (1.31-1.92)	1.46 (1.20-1.78)
**Mental co-morbidities**					
Common mental disorders[o] from three years before up to inclusion	43 (5.4)	1.25 (0.92-1.71)	0.80 (0.59-1.11)	–	0.90 (0.66-1.24)
Other mental disorders[o] from three years before up to inclusion	101 (7.6)	1.86 (1.51-2.30)	1.40 (1.13-1.74)	–	1.46 (1.17-1.82)
Psychiatric medication[q] in the year before inclusion	241 (7.5)	2.10 (1.79-2.46)	1.39 (1.16-1.66)	–	1.24 (1.03-1.48)

[a] Adjusted for sex, age, educational level, country of birth, type of living area, family situation, trajectory groups of SA/DP and previous unemployment
[b] Adjusted for sex, age, educational level, country of birth, type of living area, family situation, trajectory groups of SA/DP and previous unemployment, inpatient and specialised outpatient care due to common mental disorders and other mental disorders, and psychiatric medications; Mental co-morbidities were not mutually controlled
[c] Adjusted for sex, age, educational level, country of birth, type of living area, family situation, trajectory groups of SA/DP and previous unemployment, inpatient and specialised outpatient care due to common mental disorders and other mental disorders, and psychiatric medications, type of infarction, type of coronary revascularisation, musculoskeletal disorders, diabetes mellitus, renal insufficiency, hypertension, stroke, cancer and other somatic disorders; Mental co-morbidities were not mutually controlled
[d] Measured on 31st December of the year preceding acute myocardial infarction
[e] Missing data is considered compulsory education
[f] Missing data is considered Non-European countries
[g] Type of living area: big cities (Stockholm, Gothenburg and Malmö); medium sized cities (cities with more than 90 000 inhabitants within 30 km distance from the centre of the city); small cities/villages/rural
[h] Missing data is considered single living without children
[i] Married includes all living with partner; cohabitant
[j] Single includes divorced, separated, or widowed
[k] See method section for the International Classification of Diseases version 10 (ICD-10) codes or the Anatomic Therapeutic Chemical classification system (ATC) codes
[l] ST-elevation myocardial infarction
[m] Non-ST-elevation myocardial infarction
[n] See method section for the Classification of Surgical Procedures
[o] Measured by main or side diagnosis in inpatient or specialised outpatient care
[p] Additionally measured by prescribed antidiabetic medication
[q] Measured by antidepressants, anxiolytics and sedatives

For peer review only

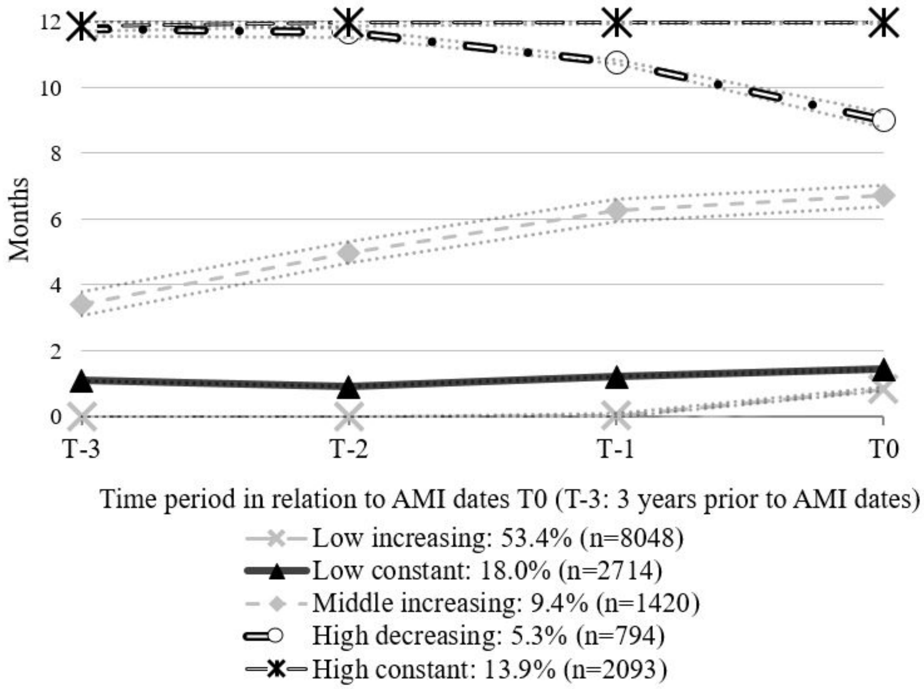

Figure 1. Trajectory groups of sickness absence and disability pension (SA/DP) months before the hospital admission date for acute myocardial infarction (AMI) in 2008-2010 (T0) and percentages of individuals in each trajectory group (n=15 069). The dotted lines represent 95% CIs.

211x150mm (96 x 96 DPI)

Reporting checklist for cohort study.

Based on the STROBE cohort guidelines.

Instructions to authors

Complete this checklist by entering the page numbers from your manuscript where readers will find each of the items listed below.

Your article may not currently address all the items on the checklist. Please modify your text to include the missing information. If you are certain that an item does not apply, please write "n/a" and provide a short explanation.

Upload your completed checklist as an extra file when you submit to a journal.

In your methods section, say that you used the STROBE cohortreporting guidelines, and cite them as:

von Elm E, Altman DG, Egger M, Pocock SJ, Gotzsche PC, Vandenbroucke JP. The Strengthening the Reporting of Observational Studies in Epidemiology (STROBE) Statement: guidelines for reporting observational studies.

		Reporting Item	Page Number
**Title and abstract**			
Title	#1a	Indicate the study's design with a commonly used term in the title or the abstract	1

For peer review only

Abstract	#1b	Provide in the abstract an informative and balanced summary of what was done and what was found	2-3
**Introduction**			
Background / rationale	#2	Explain the scientific background and rationale for the investigation being reported	5-6
Objectives	#3	State specific objectives, including any prespecified hypotheses	6
**Methods**			
Study design	#4	Present key elements of study design early in the paper	7
Setting	#5	Describe the setting, locations, and relevant dates, including periods of recruitment, exposure, follow-up, and data collection	7-8
Eligibility criteria	#6a	Give the eligibility criteria, and the sources and methods of selection of participants. Describe methods of follow-up.	7
Eligibility criteria	#6b	For matched studies, give matching criteria and number of exposed and unexposed	n/a It is not a matched study.
Variables	#7	Clearly define all outcomes, exposures, predictors, potential confounders, and effect modifiers. Give diagnostic criteria, if applicable	8-9

For peer review only

Data sources / measurement	#8	For each variable of interest give sources of data and details of methods of assessment (measurement). Describe comparability of assessment methods if there is more than one group. Give information separately for for exposed and unexposed groups if applicable.	7-9
Bias	#9	Describe any efforts to address potential sources of bias	17
Study size	#10	Explain how the study size was arrived at	7
Quantitative variables	#11	Explain how quantitative variables were handled in the analyses. If applicable, describe which groupings were chosen, and why	7-8
Statistical methods	#12a	Describe all statistical methods, including those used to control for confounding	8-9
Statistical methods	#12b	Describe any methods used to examine subgroups and interactions	8-9
Statistical methods	#12c	Explain how missing data were addressed	8-9
Statistical methods	#12d	If applicable, explain how loss to follow-up was addressed	n/a there is no loss to follow-up
Statistical methods	#12e	Describe any sensitivity analyses	9

Results

For peer review only

Participants	#13a	Report numbers of individuals at each stage of study—eg numbers potentially eligible, examined for eligibility, confirmed eligible, included in the study, completing follow-up, and analysed. Give information separately for for exposed and unexposed groups if applicable.	11
Participants	#13b	Give reasons for non-participation at each stage	n/a
Participants	#13c	Consider use of a flow diagram	n/a
Descriptive data	#14a	Give characteristics of study participants (eg demographic, clinical, social) and information on exposures and potential confounders. Give information separately for exposed and unexposed groups if applicable.	11
Descriptive data	#14b	Indicate number of participants with missing data for each variable of interest	n/a
Descriptive data	#14c	Summarise follow-up time (eg, average and total amount)	10
Outcome data	#15	Report numbers of outcome events or summary measures over time. Give information separately for exposed and unexposed groups if applicable.	11
Main results	#16a	Give unadjusted estimates and, if applicable, confounder-adjusted estimates and their precision (eg, 95% confidence interval). Make clear which confounders were adjusted for and why they were included	11-12

For peer review only

Main results	#16b	Report category boundaries when continuous variables were categorized	11-12
Main results	#16c	If relevant, consider translating estimates of relative risk into absolute risk for a meaningful time period	n/a
Other analyses	#17	Report other analyses done—e.g., analyses of subgroups and interactions, and sensitivity analyses	n/a
**Discussion**			
Key results	#18	Summarise key results with reference to study objectives	17
Limitations	#19	Discuss limitations of the study, taking into account sources of potential bias or imprecision. Discuss both direction and magnitude of any potential bias.	17
Interpretation	#20	Give a cautious overall interpretation considering objectives, limitations, multiplicity of analyses, results from similar studies, and other relevant evidence.	13-17
Generalisability	#21	Discuss the generalisability (external validity) of the study results	16
**Other Information**			
Funding	#22	Give the source of funding and the role of the funders for the present study and, if applicable, for the original study on which the present article is based	18

Notes:

- 6b: n/a It is not a matched study.

For peer review only

- 12d: n/a there is no loss to follow-up The STROBE checklist is distributed under the terms of the Creative Commons Attribution License CC-BY. This checklist was completed on 13. August 2019 using https://www.goodreports.org/, a tool made by the EQUATOR Network in collaboration with Penelope.ai

For peer review only

BMJ Open

Socio-demographic, labour market marginalisation, and medical characteristics as risk factors for re-infarction and mortality within one year after a first acute myocardial infarction-A register-based cohort study of a working age population in Sweden

Journal:	*BMJ Open*
Manuscript ID	bmjopen-2019-033616.R1
Article Type:	Original research
Date Submitted by the Author:	08-Nov-2019
Complete List of Authors:	Wang, Mo; Karolinska Institute,<br>Vaez, Marjan; Karolinska Institute, Division of Insurance Medicine, Department of Clinical Neuroscience<br>Dorner, Thomas; Medizinische Universitat Wien, Centre for Public Health, Institute of Social Medicine<br>Rahman, Syed; Karolinska Institute, Division of Insurance Medicine, Department of Clinical Neuroscience<br>Helgesson, Magnus; Karolinska Institute, Division of Insurance Medicine, Department of Clinical Neuroscience<br>Ivert, Torbjörn; Karolinska Institute, Department of Molecular Medicine and Surgery<br>Mittendorfer-Rutz, Ellenor; Karolinska Institute, Division of Insurance Medicine, Department of Clinical Neuroscience
**Primary Subject Heading**:	Epidemiology
Secondary Subject Heading:	Cardiovascular medicine
Keywords:	Acute myocardial infarction, Re-infarction, Mortality, Sick leave, Disability pension, Insurance Medicine

SCHOLARONE™
Manuscripts

Risk factors for re-infarction and mortality after acute myocardial infarction

Socio-demographic, labour market marginalisation, and medical characteristics as risk factors for re-infarction and mortality within one year after a first acute myocardial infarction-A register-based cohort study of a working age population in Sweden

Mo Wang[1], Marjan Vaez[1], Thomas Dorner[2], Syed Rahman[1], Magnus Helgesson[1], Torbjörn Ivert[3], Ellenor Mittendorfer-Rutz[1]

[1] Division of Insurance Medicine, Department of Clinical Neuroscience, Karolinska Institutet, SE 171 77 Stockholm, Sweden

[2] Department of Social and Preventive Medicine, Centre for Public Health, Medical University of Vienna, Vienna, Austria

[3] Department of Molecular Medicine and Surgery, Karolinska Institutet and Heart and Vascular Theme, Karolinska University Hospital, Stockholm, Sweden

Send requests for offprint to Corresponding author:

Mo Wang

Department of Clinical Neuroscience

Division of Insurance Medicine

Karolinska Institutet

SE-171 77 Stockholm

Sweden

E-mail: mo.wang@ki.se

Tel.: +46-8-524 832 24

Fax: +46-8-524 832 05

Word count: 3178

For peer review only - http://bmjopen.bmj.com/site/about/guidelines.xhtml

Risk factors for re-infarction and mortality after acute myocardial infarction

Abstract

Objectives: Research covering a wide range of risk factors related to the prognosis during the first year after an acute myocardial infarction (AMI) is insufficient. This study aimed to investigate whether socio-demographic, labour market marginalisation, and medical characteristics before/at AMI were associated with subsequent re-infarction and all-cause mortality.

Design: Population-based cohort study.

Participants: The cohort included 15 069 individuals aged 25-64 years who had a first AMI during 2008-2010.

Primary and secondary outcome measures: The outcome measures consisted of re-infarction and all-cause mortality within one year following an AMI, which were estimated by univariate and multivariable hazard ratios (HR) and 95% confidence intervals (CI) by Cox regression.

Results: Socio-demographic characteristics such as lower education showed a 1.1- and 1.3-fold higher risk for re-infarction and mortality, respectively. Older age was associated with a higher risk of mortality while being born in non-European countries showed a lower risk of mortality. Labour market marginalisation such as previous long-term work disability was associated with a 2-fold higher risk of mortality. Regarding medical characteristics, ST-elevation myocardial infarction was predictive for re-infarction (HR: 1.14, 95% CI: 1.07-1.21) and all-cause mortality (HR: 3.80, 95% CI: 3.08-4.68). Moreover, diabetes mellitus, renal insufficiency, stroke, cancer, and mental disorders were associated with a higher risk of mortality (range of HRs: 1.24-2.59).

Conclusions: Socio-demographic and medical risk factors were identified as risk factors for mortality and re-infarction after AMI, including older age, immigration status, somatic and mental co-morbidities. Previous long-term work disability and infarction type provide useful

For peer review only - http://bmjopen.bmj.com/site/about/guidelines.xhtml

Risk factors for re-infarction and mortality after acute myocardial infarction

information for predicting adverse outcomes after AMI during the first year, particularly for mortality.

Keywords: Acute myocardial infarction; Re-infarction; Mortality; Sick leave; Disability pension; Insurance Medicine.

For peer review only

Risk factors for re-infarction and mortality after acute myocardial infarction

ARTICLE SUMMARY

Strengths and limitations of this study

- This is a population-based cohort study on all patients with acute myocardial infarction from inpatient care.
- The Swedish national-wide register data has high quality, which reduces the risk of recall bias regarding exposure and outcome.
- Despite a wide range of risk factors that have been examined, some potential for residual confounding by unmeasured factors remains.
- There is no available information on sick-leave spells that are shorter than 14 days among employed individuals.

For peer review only

Risk factors for re-infarction and mortality after acute myocardial infarction

Introduction

Acute myocardial infarction (AMI), is the leading cause of mortality worldwide and re-infarction is common, ranging from 8% to 20% in the first year (1). Over the past decade, percutaneous coronary intervention (PCI) and medication have reduced mortality in AMI patients (2, 3). Despite this progress, AMI remains a major cause of mortality and disability. For patients who survive a first AMI, post-discharge optimal medical management and healthy life-style are essential. Particularly, re-infarction and heart failure can occur after an AMI, influencing quality of life and increasing healthcare costs (1, 4, 5). Knowledge of risk factors for re-infarction and mortality in the first year after an AMI could improve the ability of healthcare providers to reduce progression of disease as well as improve survival after AMI.

Previous studies have reported risk factors for re-infarction and mortality in patients with AMI, mainly focusing on events within the first month after discharge (6). Socio-demographic characteristics such as older age, lower socio-economic status, living alone, and (co-)morbidity (e.g. diabetes mellitus, renal diseases, hypertension, unstable angina, stroke or transient ischemic attack, cancer, and depression) have been found to be associated with a higher risk of re-infarction and mortality after discharge (6-10). None of these studies have taken into consideration risk factors for re-infarction or mortality in the mid-term i.e. one year after hospital discharge. Moreover, currently there is little evidence related to crucial AMI-related characteristics such as type of coronary revascularisation and infarction. Here, studies are lacking which include a vast range of risk factors and are based on register data, which provide large study populations and guarantee practically no loss to follow-up.

For peer review only - http://bmjopen.bmj.com/site/about/guidelines.xhtml

Additionally, there is a lack of studies elucidating the associations between characteristics of labour market marginalisation and the risk of re-infarction and mortality among AMI patients. In Sweden, more than 30 000 persons experience an AMI each year; of these, about 10 000 are below the age of 65 (11). This burden of disease may result in long-term work disability in the working age population (12, 13). To date, sickness absence (SA) is almost always prescribed as a rehabilitation strategy in healthcare services for patients with AMI (14). Also, permanent work disability, i.e. disability pension (DP), is common in this patient group (12). In a prior study, patterns of SA/DP before AMI provided crucial information for subsequent work disability (15). To the best of our knowledge, this is the first study investigating labour market marginalisation measured in terms of trajectories of SA/DP and unemployment status as risk factors for re-infarction and mortality in patients with AMI.

Aims

The study aimed to investigate to what extent socio-demographic, labour market marginalisation, and medical (including AMI-related factors and co-morbidities) characteristics before/at an AMI were associated with subsequent re-infarction and all-cause mortality during the first year.

For peer review only - http://bmjopen.bmj.com/site/about/guidelines.xhtml

Materials and Methods

Study population

This is a nationwide register-based cohort study and the study population consisted of 16 983 individuals aged 25 to 64 who had a first AMI during 2008-2010. A main diagnosis of AMI was ascertained from the inpatient care register and defined according to the International Classification of Diseases (ICD)-10 code of I21. This means that individuals with a previous main or side diagnosis of AMI in specialised healthcare from 1987 up to the hospital admission date for AMI during 2008-2010 were excluded (n=1914). Altogether, there were 15 069 individuals included in the study.

Registers

National register data was linked to the study population by using the unique personal identity number assigned to all Swedish inhabitants, including information for each individual up to 31st December 2013 from:

1.) Statistics Sweden: sex, age, education, country of birth, type of living area, family situation, length of unemployment, and year of emigration from the Longitudinal integration database for health insurance and labour market studies (LISA);

2.) The Social Insurance Agency: SA/DP (date and grade) from Micro-data for analyses of social insurance (MiDAS);

3.) The National Board of Health and Welfare: date and cause of diagnosis-specific inpatient and specialised outpatient care, and type of infarction and type of coronary revascularisation from the National Patient Register; date of death from the Cause of Death Register (16) and date, type and dose of prescription of dispensed psychiatric medication and antidiabetic medication from the National Prescribed Drug Register.

For peer review only - http://bmjopen.bmj.com/site/about/guidelines.xhtml

Outcome measures

The outcome measures were re-infarction (ICD-codes: I21) which was ascertained from the inpatient care, and all-cause mortality during the first year after AMI.

Risk measures

Socio-demographic characteristics were recorded at the end of the year preceding AMI and comprised: sex, age, education (low educational level (compulsory (≤9 years)), high school (10-12 years), and high educational level (university (>12 years))), country of birth, type of living area, and family situation (Table 1).

Labour market marginalisation characteristics included length of unemployment in the year preceding AMI and the trajectory groups of SA/DP during three years before and up to the AMI diagnosis (Table 1). The trajectory groups of SA/DP were measured using the combined mean number of annual SA and DP net days before the AMI diagnosis. The total number of net days were then transformed to number of months with SA/DP.

Medical characteristics included AMI-related characteristics (type of infarction and type of coronary revascularisation) at inclusion and inpatient and specialised outpatient care due to any main or side diagnosis of somatic and mental co-morbidities and medication which were measured from three years before until the AMI diagnosis. Type of infarction was classified as ST-elevation myocardial infarction (STEMI, ICD-codes: I21.0-I21.3), non-ST-elevation myocardial infarction (NSTEMI, ICD-codes: I21.4), or unspecified (ICD-codes: I21.9). Information on type of coronary revascularisation was categorised as: percutaneous coronary intervention (PCI) (FNG00-FNG05), coronary artery bypass grafting (CABG) (FNA-FNF, FNG30, FNW96), and others (i.e. other treatments/examinations or missing information).

For peer review only - http://bmjopen.bmj.com/site/about/guidelines.xhtml

Risk factors for re-infarction and mortality after acute myocardial infarction

Somatic co-morbidities were categorised as musculoskeletal diagnoses (ICD-codes: M00-99), renal insufficiency (ICD-codes: N17-N19), stroke (ICD-codes: I60, I61, I63, I64), hypertension (ICD-codes: I10), cancer (ICD-codes: C00-D48), and other somatic disorders (the other ICD-codes except for mental diagnoses). The individuals with any specialised care due to diabetes mellitus or having any prescribed antidiabetic medication were coded according to ICD-codes: E10-E14 and the Anatomic Therapeutic Chemical classification system (ATC) code: A10. Mental co-morbidities were grouped as CMDs (i.e. depressive (ICD-codes: F32-F33), anxiety (ICD-codes: F40-F42) and stress-related disorders (ICD-codes: F43)), and other mental disorders (ICD-codes: F00-F31, F34-F39, and F44-F99). Moreover, prescribed psychiatric medication during the year preceding the AMI diagnosis was included as mental co-morbidities. Psychiatric medication was measured by any antidepressants, anxiolytics and sedatives following the ATC codes, N06A, N05B and N05C, respectively.

Statistical analyses

We used group-based trajectory modelling to estimate groups of SA/DP trajectories during the 3-year period before AMI. This method has been described elsewhere (15, 17). Five groups were selected as the best fitting model for patients with AMI. An annual time-scale was used in the study, where T0 represents the first hospital admission date due to AMI and T-3 represents the 3 years before the first AMI diagnosis (See Fig. 1). The five trajectory groups were named according to the patterns of each group: “Low increasing”, “Low constant”, “Middle increasing”, “High decreasing” and “High constant”.

For peer review only - http://bmjopen.bmj.com/site/about/guidelines.xhtml

185 Chi-2 tests were used to estimate potential sex differences regarding all the examined characteristics among patients with AMI. Hazard ratios (HRs) and 95% confidence intervals (CIs) for re-infarction and all-cause mortality were calculated using Cox regression. The proportional hazards assumption was tested and met. Follow-up time started from the first hospital admission date due to AMI diagnosis until the events (re-infarction or all-cause mortality), emigration to a foreign country, or the end of the first year after AMI, whichever came first. Mean follow-up time for re-infarction and all-cause mortality was 117 days (SD 120) and 177 days (SD 109), respectively. Interaction analyses were performed for sex and age, however, no interaction effects were found. We also carried out a sensitivity analysis with mortality due to cardiovasular diseases as outcome measure (See supplementary table). Analyses were adjusted for all risk measures in the multivariate model (mental co-morbidities were not mutually adjusted). Data processing was performed using SAS version 9.4.

Patient and public involvement

There was no patient involvement in this study.

For peer review only - http://bmjopen.bmj.com/site/about/guidelines.xhtml

Results

Table 1 shows descriptive analysis for patients with a first AMI during 2008-2010. Of all, there were 3673 women (24.4%). The majority of the study population was older (56-64 years, 59.3%), born in Sweden (80.2%), belonged to the low increasing SA/DP trajectory group (53.4%) (Fig. 1), were not unemployed before inclusion (91.6%), received PCI at inclusion (68.8%), had other somatic disorders (67.1%), and did not have mental co-morbidities. Re-infarction and all-cause mortality during the first year represented 35.2% and 4.4% of the study population, respectively. Futhermore, sex differences were significant for various factors. For example, with respect to labour market marginalisation characteristics, the "Low increasing" SA/DP group comprised more men (58.8% vs. 36.6%) while the "High constant" SA/DP group was more common for women (22.7% vs. 11.1%). Moreover, more men had a STEMI (36.9% vs. 28.8%) and received a PCI (72.5% vs. 57.2%) compared to women while more women had co-morbidities compared to men.

Re-infarction

In the univariate analyses, higher risks of re-infarction were found in those with lower education and living in small towns/villages (data not shown). In contrast, those born in non-Nordic European countries, and those living in medium-sized cities had lower risks of subsequent re-infarction during the first year. Moreover, a higher risk of re-infarction was observed among those with STEMI compared to non-STEMI as well as those treated with CABG compared to PCI (HR 2.43; 95% CI 2.14-2.75) (Table 2).

In the final model, lower educational level and living in small towns/villages were associated with a higher risk of re-infarction while living in medium-sized cities, and being single living

For peer review only - http://bmjopen.bmj.com/site/about/guidelines.xhtml

without children at home showed lower risk of re-infarction. With regard to AMI-related characteristics, patients with STEMI and CABG had a higher risk of re-infarction (Table 2).

All-cause mortality

In the multivariable model, we found that older age, lower level of education, being married/single living without children at home, and belonging to the "High constant" SA/DP trajectory group were risk factors for all-cause mortality during the first year after AMI. Those born in non-European countries and those belonging to the "Low constant "and "High decreasing" SA/DP trajectory groups were associated with a lower risk of all-cause mortality. STEMI compared to non-STEMI was associated with a 4-fold higher risk of all-cause mortality following AMI. Moreover, a higher risk of all-cause mortality was found in patients with diabetes mellitus, renal insufficiency, stroke, cancer and other somatic disorders compared to those without such co-morbidities. Other mental disorders besides CMDs and psychiatric medication were significantly associated with subsequent all-cause mortality (Table 3). The sensitivity analysis with mortality due to cardiovascular diseases as the outcome showed similar results as for all-cause mortality (See supplementary table).

For peer review only - http://bmjopen.bmj.com/site/about/guidelines.xhtml

DISCUSSION

Socio-demographic and labour market marginalisation

Socio-demographic and labour market marginalisation were generally more associated with mortality than re-infarction in AMI patients. For instance, results showed that a lower education level, which acts as a proxy of lower socioeconomic status, was associated with a less favourable prognosis regarding re-infarction (HR: 1.12) and all-cause mortality (HR: 1.29) during the first year after AMI. Previous studies have shown that patients with a lower educational level generally have a higher risk profile, primarily due to the presence of more risk factors such as smoking or the resistance of quitting smoking after AMI and co-morbidities, leading to a worse health outcome (18-20). After adjustment for co-morbidities, we found that educational level remained an independent predictor of re-infarction and mortality, Still, one cannot rule out the possibility of unmeasured residual co-morbidities that may be associated with re-infarction and all-cause mortality.

As expected, we observed that higher age was a strong predictor of all-cause mortality after AMI, which is in agreement with other studies (21-23). Somewhat unexpectedly, older age was not associated with re-infarction during the first year. The different findings with respect to mortality and re-infarction may be driven by the co-morbidities that were controlled for in the model, which are closely related with AMI and the association between age and all-cause mortality might be caused by other co-morbidites. Interestingly, we found a higher risk of re-infarction for patients who were living in small towns/villages, while a lower risk of re-infarction was observed for those living in medium sized cities compared to those living in big cities. This result might indicate diversities in healthcare in relation to different types of living area (24).

For peer review only - http://bmjopen.bmj.com/site/about/guidelines.xhtml

Furthermore, AMI patients born in non-European countries had a lower risk of all-cause mortality during the first year than patients born in Sweden. Recent research has shown a lower risk of mortality after AMI among South Asians compared with the host population (25, 26). Our finding may also reflect a "healthy migrant effect", indicating a positive health selection of migrants who are able to overcome the obstacles of migration. Previous studies showed that migrants have revealed a lower risk of morbidity and mortality compared to natives (27, 28).

Compared to AMI patients who were married and living with children at home, those who were married/single and living without children at home had a higher risk of all-cause mortality. Patients who live alone may have poor adherence to medication and follow-up recommendations, which might be associated with an unfavourable outcome. The few studies that have described the association between social support and prognosis in patients with coronary artery disease have had inconsistent definitions of measures of social support, leading to a wide variety of conclusions (29). Therefore, the impact of family situation on re-infarction and all-cause mortality is open to speculation and warrants further investigation.

With regard to labour market marginalisation factors, the "High constant" SA/DP trajectory group was associated with a 2.2-fold higher risk of all-cause mortality, even after controlling for confounders. As this group had around 12 months of SA/DP per annum before AMI, it is likely that this group consisted of a larger proportion of individuals with long-term SA or DP. This group may also have had a history of co-morbidities before AMI, which in turn increases the risk of all-cause mortality. On the other hand, the "Low constant" and "High decreasing" SA/DP trajectory groups showed a lower risk of all-cause mortality after adjusting for co-morbidities. Our study is the first to report that SA/DP trajectory groups can be used as risk

For peer review only

For peer review only - http://bmjopen.bmj.com/site/about/guidelines.xhtml

factors for mortality in patients with AMI. Our findings also revealed that risk estimates of SA/DP trajectory groups were comparable to well-known risk factors such as diabetes mellitus and renal insufficiency. Therefore, more attention in clinical practice in relation to work disabilityfactors in AMI patients is necessary.

Medical characteristics

Patients with co-morbidities and STEMI had a higher risk of adverse outcomes, particularly for all-cause mortality, while those who underwent CABG had a higher risk of re-infarction. Indeed, STEMI is clinically associated with more serious medical conditions than non-STEMI (30). With respect to coronary revascularisation, some studies have found that patients treated with PCI rather than CABG had fewer complications and a lower risk of mortality, particularly in the short-term (31, 32).

In addition, a higher risk of all-cause mortality was observed among patients with mental co-morbidities. Both biological and behavioural mechanisms have been suggested to explain the association between mental disorders and cardiovascular disease. Patients with mental disorders have been reported to have several cardiac symptoms (33, 34). Further, they tend to have poorer diets, reduced medication adherence, and more stress (35). Overall, mental disorders reduce the success of interventions targeting cardiovascular risk factor modification, leading to higher healthcare costs, poorer health outcomes, and increased mortality rates.

Strengths and limitations

The strengths of this study include the use of a population-based cohort design, which offers satisfactory statistical power for the analyses. The use of high quality national register data

For peer review only - http://bmjopen.bmj.com/site/about/guidelines.xhtml

also minimises the risk of recall bias regarding exposure and outcome (36, 37). There still might be misclassification and missing information in the register data. However, misclassification and missing information seems to be randomly distributed across the different exposure and outcome measures and this misclassification is assumed to be non-differential. The high coverage of the register data also enabled us to identify all AMI patients from inpatient care with subsequent re-infarction and mortality. We included only AMI patients who were treated in inpatient care with more severe cardiac disease. This might explain the high incidence of re-infarction during the first year of the study. We also used an advanced method covering the inherent heterogeneity, group-based trajectory modelling, to investigate work disability patterns in the study. Moreover, we were able to examine a wide range of risk factors as well as adjust for relevant confounders. Still, there might be other factors than those studied here that are associated with re-infarction and mortality. Our registers did not include information of compliance to prescribed medication such as dual-antiplatelet therapy, smoking habits before and after AMI, rehabilitation measures and life-style changes.

Limitations of the study and considerations when interpreting our findings are acknowledged. In this study, we only included co-morbidities recorded in inpatient and specialised outpatient care, but not those from primary care due to lack of data availability. While we adjusted for potential confounders that were particularly relevant for AMI, we acknowledge that there may be a wider range of co-morbidities that we were unable to control for. Mental co-morbidities were measured by including prescribed psychiatric medication data. For somatic co-morbidities, we did not include an equivalent measure except for diabetes mellitus as there was no available information in the register data. With regard to sickness absence, we did not have information on sick-leave spells that were less than 14 days among employed

For peer review only - http://bmjopen.bmj.com/site/about/guidelines.xhtml

individuals. Thus, the number of SA days contributing to the combined number of SA/DP days might be underestimated.

Conclusions

Several socio-demographic and co-morbidity risk factors were generally associated more strongly with mortality than re-infarction in AMI patients, including lower educational level, older age, immigration status, somatic and mental co-morbidities. Previous long-term work disability and infarction type showed a higher risk for all-cause mortality after AMI during the first year.

For peer review only

Author Contributions

EM, MV and MW conceived and designed the study. MW and EM were involved in the statistical analysis and drafted the manuscript. MW, MV, TD, SR, MH, TI and EM contributed to the critical revision and approved the manuscript.

Acknowledgements

None.

Funding

This study was supported by the Swedish Research Council, grant nr 2015-02292.

Declaration of conflicting interests

None.

ETHICS APPROVAL

The study has been evaluated and approved by the Regional Ethical Review Board of Karolinska Institutet, Stockholm, Sweden (2007/762–31). The ethical review board approved the study and waived the requirement that informed consent of research subjects should be obtained.

DATA SHARING STATEMENT

The data that support the findings of this study are available from Statistics Sweden and The Swedish National Board of Health and Welfare, but restrictions apply to the availability of these data, which were used with ethical permission for the current study and therefore are not publicly available.

For peer review only

Risk factors for re-infarction and mortality after acute myocardial infarction

References

1. Fiscella K, Meldrum S, Barnett S. Hospital discharge against advice after myocardial infarction: deaths and readmissions. The American journal of medicine. 2007;120(12):1047-53.
2. Mohr FW, Morice MC, Kappetein AP, Feldman TE, Stahle E, Colombo A, et al. Coronary artery bypass graft surgery versus percutaneous coronary intervention in patients with three-vessel disease and left main coronary disease: 5-year follow-up of the randomised, clinical SYNTAX trial. Lancet. 2013;381(9867):629-38.
3. Chung SC, Gedeborg R, Nicholas O, James S, Jeppsson A, Wolfe C, et al. Acute myocardial infarction: a comparison of short-term survival in national outcome registries in Sweden and the UK. Lancet (London, England). 2014;383(9925):1305-12.
4. De Smedt D, Annemans L, De Backer G, Kotseva K, Ryden L, Wood D, et al. Cost-effectiveness of optimized adherence to prevention guidelines in European patients with coronary heart disease: Results from the EUROASPIRE IV survey. International journal of cardiology. 2018;272:20-5.
5. De Smedt D, Kotseva K, De Bacquer D, Wood D, De Backer G, Dallongeville J, et al. Cost-effectiveness of optimizing prevention in patients with coronary heart disease: the EUROASPIRE III health economics project. European heart journal. 2012;33(22):2865-72.
6. Dunlay SM, Weston SA, Killian JM, Bell MR, Jaffe AS, Roger VL. Thirty-day rehospitalizations after acute myocardial infarction: a cohort study. Annals of internal medicine. 2012;157(1):11-8.
7. Khawaja FJ, Shah ND, Lennon RJ, Slusser JP, Alkatib AA, Rihal CS, et al. Factors associated with 30-day readmission rates after percutaneous coronary intervention. Archives of internal medicine. 2012;172(2):112-7.
8. Kamalesh M, Subramanian U, Ariana A, Sawada S, Tierney W. Similar decline in post-myocardial infarction mortality among subjects with and without diabetes. The American journal of the medical sciences. 2005;329(5):228-33.
9. Brown JR, Conley SM, Niles NW, 2nd. Predicting readmission or death after acute ST-elevation myocardial infarction. Clinical cardiology. 2013;36(10):570-5.
10. Agrawal Y, Panaich S, Aggarwal S, Saltiel F, Kalavakunta JK, Gupta V. Demographic parameters related to 30-day readmission of patients with acute myocardial infarction: Analysis of 2,371,867 hospitalizations. International journal of cardiology. 2016;214:408-9.
11. Annual report of SWEDEHEART 2010 (Årsrapport SWEDEHEART 2010. RIKS-HIA, SEPHIA, SCAAR, Hjärtkirurgi och TAVI. In Swedish) [Available from: http://www.ucr.uu.se/swedeheart/dokument-sh/arsrapporter/aeldre-arsrapporter-older-reports/arsrapporter-2007-2010.
12. Zetterstrom K, Vaez M, Alexanderson K, Ivert T, Pehrsson K, Hammar N, et al. Disability pension after coronary revascularization: a prospective nationwide register-based Swedish cohort study. European journal of preventive cardiology. 2015;22(3):304-11.
13. Smedegaard L, Nume AK, Charlot M, Kragholm K, Gislason G, Hansen PR. Return to Work and Risk of Subsequent Detachment From Employment After Myocardial Infarction: Insights From Danish Nationwide Registries. Journal of the American Heart Association. 2017;6(10).
14. National Board of Health and Welfare (NBHW, Socialstyrelsen): Basis for descision ininsurance medicine (försäkringsmedicinskt beslutstöd) 2015 [Available from: http://www.socialstyrelsen.se/riktlinjer/forsakringsmedicinsktbeslutsstod.
15. Wang M, Vaez M, Dorner TE, Tiihonen J, Voss M, Ivert T, et al. Trajectories and characteristics of work disability before and after acute myocardial infarction. Heart. 2017.

For peer review only - http://bmjopen.bmj.com/site/about/guidelines.xhtml

16. Socialstyrelsen. Dödsorsaksregistret, Patientregistret. [Cause of Death register, National Patient Register; online resource, in Swedish]. . Stockholm: National Board of Health and Welfare; 2009.
17. Jones B, Nagin D, Roeder K. A SAS Procedure Based on Mixture Models for Estimating Developmental Trajectories. Sociological Methods & Research 2001;29 (3):374-93.
18. Mehta RH, O'Shea JC, Stebbins AL, Granger CB, Armstrong PW, White HD, et al. Association of mortality with years of education in patients with ST-segment elevation myocardial infarction treated with fibrinolysis. Journal of the American College of Cardiology. 2011;57(2):138-46.
19. Lee KL, Woodlief LH, Topol EJ, Weaver WD, Betriu A, Col J, et al. Predictors of 30-day mortality in the era of reperfusion for acute myocardial infarction. Results from an international trial of 41,021 patients. GUSTO-I Investigators. Circulation. 1995;91(6):1659-68.
20. Bruthans J, Mayer O, Jr., De Bacquer D, De Smedt D, Reiner Z, Kotseva K, et al. Educational level and risk profile and risk control in patients with coronary heart disease. European journal of preventive cardiology. 2016;23(8):881-90.
21. Waldecker B, Waas W, Haberbosch W, Voss R, Heizmann H, Tillmanns H. Long-term follow-up after direct percutaneous transluminal coronary angioplasty for acute myocardial infarction. Journal of the American College of Cardiology. 1998;32(5):1320-5.
22. Califf RM, Pieper KS, Lee KL, Van De Werf F, Simes RJ, Armstrong PW, et al. Prediction of 1-year survival after thrombolysis for acute myocardial infarction in the global utilization of streptokinase and TPA for occluded coronary arteries trial. Circulation. 2000;101(19):2231-8.
23. Lee CH, van Domburg RT, Hoye A, Lemos PA, Tanabe K, Smits PC, et al. Predictors of survival after contemporary percutaneous coronary revascularization for acute myocardial infarction in the real world. The Journal of invasive cardiology. 2004;16(11):627-31.
24. Caldwell JT, Ford CL, Wallace SP, Wang MC, Takahashi LM. Intersection of Living in a Rural Versus Urban Area and Race/Ethnicity in Explaining Access to Health Care in the United States. American journal of public health. 2016;106(8):1463-9.
25. Bansal N, Fischbacher CM, Bhopal RS, Brown H, Steiner MF, Capewell S. Myocardial infarction incidence and survival by ethnic group: Scottish Health and Ethnicity Linkage retrospective cohort study. BMJ open. 2013;3(9):e003415.
26. Zaman MJ, Philipson P, Chen R, Farag A, Shipley M, Marmot MG, et al. South Asians and coronary disease: is there discordance between effects on incidence and prognosis? Heart (British Cardiac Society). 2013;99(10):729-36.
27. Di Thiene D, Alexanderson K, Tinghog P, La Torre G, Mittendorfer-Rutz E. Suicide among first-generation and second-generation immigrants in Sweden: association with labour market marginalisation and morbidity. J Epidemiol Community Health. 2015;69(5):467-73.
28. Guillot M, Khlat M, Elo I, Solignac M, Wallace M. Understanding age variations in the migrant mortality advantage: An international comparative perspective. PloS one. 2018;13(6):e0199669.
29. Case RB, Moss AJ, Case N, McDermott M, Eberly S. Living alone after myocardial infarction. Impact on prognosis. Jama. 1992;267(4):515-9.
30. Anderson JL, Morrow DA. Acute Myocardial Infarction. The New England journal of medicine. 2017;376(21):2053-64.
31. Stone GW, White HD, Ohman EM, Bertrand ME, Lincoff AM, McLaurin BT, et al. Bivalirudin in patients with acute coronary syndromes undergoing percutaneous coronary intervention: a subgroup analysis from the Acute Catheterization and Urgent Intervention Triage strategy (ACUITY) trial. Lancet (London, England). 2007;369(9565):907-19.

For peer review only - http://bmjopen.bmj.com/site/about/guidelines.xhtml

32. Hochholzer W, Buettner HJ, Trenk D, Breidthardt T, Noveanu M, Laule K, et al. Percutaneous coronary intervention versus coronary artery bypass grafting as primary revascularization in patients with acute coronary syndrome. The American journal of cardiology. 2008;102(2):173-9.
33. Sherwood A, Hinderliter AL, Watkins LL, Waugh RA, Blumenthal JA. Impaired endothelial function in coronary heart disease patients with depressive symptomatology. Journal of the American College of Cardiology. 2005;46(4):656-9.
34. Empana JP, Sykes DH, Luc G, Juhan-Vague I, Arveiler D, Ferrieres J, et al. Contributions of depressive mood and circulating inflammatory markers to coronary heart disease in healthy European men: the Prospective Epidemiological Study of Myocardial Infarction (PRIME). Circulation. 2005;111(18):2299-305.
35. Lett HS, Blumenthal JA, Babyak MA, Sherwood A, Strauman T, Robins C, et al. Depression as a risk factor for coronary artery disease: evidence, mechanisms, and treatment. Psychosomatic medicine. 2004;66(3):305-15.
36. Ludvigsson JF, Andersson E, Ekbom A, Feychting M, Kim JL, Reuterwall C, et al. External review and validation of the Swedish national inpatient register. BMC public health. 2011;11:450.
37. Brooke HL, Talback M, Hornblad J, Johansson LA, Ludvigsson JF, Druid H, et al. The Swedish cause of death register. European journal of epidemiology. 2017;32(9):765-73.

For peer review only

For peer review only - http://bmjopen.bmj.com/site/about/guidelines.xhtml

Risk factors for re-infarction and mortality after acute myocardial infarction

Table 1: Descriptive statistics for all women (n=3673) and men (n=11 396) aged between 25 and 64 years with a diagnosis of acute myocardial infarction (AMI) from inpatient care in 2008-2010 in Sweden (N=15 069)

Characteristics of patients with AMI	All		Women		Men		Chi^2 (p-value)
	n 15 069	% 100	n 3673	% 24.4	n 11 396	% 75.6	
**Socio-demographic characteristics[a]**							
Age[*]							
25-45	1401	9.3	335	9.1	1066	9.4	15.1 (<0.001)
46-55	4739	31.5	1065	29.0	3674	32.2	
56-64	8929	59.3	2273	61.9	6656	58.4	
Education (years)[*b]							
Compulsory (≤9)	4474	29.7	1040	28.3	3434	30.1	9.9 (<0.01)
High school (10–12)	7435	49.3	1895	51.6	5540	48.6	
University (>12)	3160	21.0	738	20.1	2422	21.3	
Country of birth[*c]							
Sweden	12 085	80.2	2991	81.4	9094	79.8	86.4 (<0.001)
Other Nordic countries	860	5.7	282	7.7	578	5.1	
Europe (except Nordic countries)	440	2.9	116	3.2	324	2.8	
Non-European countries	1684	11.2	284	7.7	1400	12.3	
Type of living area[*d]							
Big cities	4566	30.3	1052	28.6	3514	30.8	6.5 (<0.05)
Medium sized cities	5347	35.5	1344	36.6	4003	35.1	
Small towns/villages	5156	34.2	1277	34.8	3879	34.0	
Family situation[*e]							
Married[f] living without children	4880	32.4	1342	36.5	3538	31.1	235.8 (<0.001)
Married[f] living with children	4000	26.5	728	19.8	3272	28.7	
Single[g] living without children	5386	35.7	1271	34.6	4115	36.1	
Single[g] living with children	803	5.3	332	9.0	471	4.1	
**Labour market marginalisation characteristics**							
Trajectory groups of SA/DP[*] ***from three years before up to inclusion***							
Low increasing	8048	53.4	1345	36.6	6703	58.8	705.4 (<0.001)
Low constant	2714	18.0	709	19.3	2005	17.6	
Middle increasing	1420	9.4	455	12.4	965	8.5	
High decreasing	794	5.3	331	9.0	463	4.1	
High constant	2093	13.9	833	22.7	1260	11.1	
Unemployment[*] ***in the year before inclusion***							
No unemployment	13 799	91.6	3420	93.1	10 379	91.1	15.0 (<0.001)
1-180 days	852	5.7	171	4.7	681	6.0	
>180 days	418	2.8	82	2.2	336	3.0	
**AMI-related characteristics**							
Type of infarction[*h] ***at inclusion***							
STEMI[i]	5260	34.9	1058	28.8	4202	36.9	84.7 (<0.001)
Non-STEMI[j]	6704	44.5	1832	49.9	4872	42.8	

For peer review only

For peer review only - http://bmjopen.bmj.com/site/about/guidelines.xhtml

Unspecified	3105	20.6	783	21.3	2322	20.4	
Coronary revascularisation characteristics[*k] at inclusion							
Percutaneous coronary intervention	10 364	68.8	2100	57.2	8264	72.5	353.9 (<0.001)
Coronary artery bypass grafting	336	2.2	59	1.6	277	2.4	
Others	4369	29.0	1514	41.2	2855	25.1	
**Co-morbidity characteristics[h]**							
**Somatic co-morbidities[l] from three years before up to inclusion**							
Musculoskeletal disorders[*]	2299	15.3	741	20.2	1558	13.7	90.9 (<0.001)
Diabetes mellitus[*m]	2529	16.8	675	18.4	1854	16.3	8.8 (<0.01)
Renal insufficiency	248	1.7	70	1.9	178	1.6	2.0 (0.15)
Hypertension[*]	5110	33.9	1365	37.2	3745	32.9	22.9 (<0.001)
Stroke	199	1.3	54	1.5	145	1.3	0.8 (0.36)
Cancer[*]	933	6.2	303	8.3	630	5.5	35.4 (<0.001)
Other somatic disorders[*]	10 107	67.1	2722	74.1	7385	64.8	108.9 (<0.001)
**Mental co-morbidities**							
Common mental disorders[*l] from three years before up to inclusion	791	5.3	287	7.8	504	4.4	64.2 (<0.001)
Other mental disorders[l] from three years before up to inclusion	1331	8.8	328	8.9	1003	8.8	0.1 (0.81)
Psychiatric medication[*n] in the year before inclusion	3231	21.4	1299	35.4	1932	16.7	559.1 (<0.001)
**Re-infarction and all-cause mortality during first year after AMI**							
Re-infarction	5310	35.2	1276	34.7	4034	35.4	0.5 (0.47)
All-cause mortality[*]	666	4.4	191	5.2	475	4.2	7.0 (<0.01)

[*] Significant sex differences
[a] Measured on 31st December of the year preceding acute myocardial infarction
[b] Missing data is considered compulsory education
[c] Missing data is considered Non-European countries
[d] Type of living area: big cities (Stockholm, Gothenburg and Malmö); medium sized cities (cities with more than 90 000 inhabitants within 30 km distance from the centre of the city); small cities/villages/rural
[e] Missing data is considered single living without children
[f] Married includes all living with partner; cohabitant
[g] Single includes divorced, separated, or widowed
[h] See method section for the International Classification of Diseases version 10 (ICD-10) codes or the Anatomic Therapeutic Chemical classification system (ATC) codes
[i] ST-elevation myocardial infarction
[j] Non-ST-elevation myocardial infarction
[k] See method section for the Classification of Surgical Procedures
[l] Measured by main or side diagnosis in inpatient or specialised outpatient care
[m] Additionally measured by prescribed antidiabetic medication
[n] Measured by antidepressants, anxiolytics and sedatives

For peer review only

Risk factors for re-infarction and mortality after acute myocardial infarction

Table 2. Adjusted hazard ratios (HR) and 95% Confidence Interval (CI) for re-infarction in individuals with a diagnosis of acute myocardial infarction (AMI) from inpatient care in 2008-2010 in Sweden (N=15 069) during the first year after AMI

Characteristics of patients with AMI	**Re-infarction**	Model 1[a]	Model 2[b]	Model 3[c]
	n (%)	HR (95% CI)		
**Socio-demographic characteristics[d]**				
Sex				
Men	4034 (35.4)	1	1	1
Women	1276 (34.7)	0.97 (0.91-1.04)	0.98 (0.91-1.04)	1.03 (0.97-1.11)
Age				
25-45	482 (34.4)	1	1	1
46-55	1679 (35.4)	1.02 (0.92-1.13)	1.02 (0.92-1.13)	1.01 (0.91-1.12)
56-64	3149 (35.3)	0.98 (0.89-1.09)	0.99 (0.90-1.10)	0.99 (0.90-1.10)
Education (years)[e]				
Compulsory (≤9)	1630 (36.4)	1.13 (1.05-1.23)	1.13 (1.04-1.23)	1.12 (1.04-1.22)
High school (10–12)	2672 (35.9)	1.11 (1.03-1.19)	1.11 (1.03-1.19)	1.10 (1.02-1.18)
University (>12)	1008 (31.9)	1	1	1
Country of birth[f]				
Sweden	4367 (36.1)	1	1	1
Other Nordic countries	309 (35.9)	1.01 (0.90-1.13)	1.01 (0.90-1.13)	1.02 (0.91-1.14)
Europe (except Nordic countries)	124 (28.2)	0.83 (0.69-0.99)	0.83 (0.69-0.99)	0.83 (0.70-1.00)
Non-European countries	510 (30.3)	0.91 (0.82-1.00)	0.91 (0.82-1.00)	0.91 (0.82-1.00)
Type of living area[g]				
Big cities	1368 (30.0)	1	1	1
Medium sized cities	1428 (26.7)	0.84 (0.78-0.91)	0.84 (0.78-0.91)	0.84 (0.78-0.91)
Small towns/villages	2514 (48.8)	1.81 (1.69-1.94)	1.81 (1.69-1.94)	1.83 (1.71-1.96)
Family situation[h]				
Married[i] living without children	1782 (36.5)	0.97 (0.90-1.05)	0.97 (0.90-1.05)	0.97 (0.90-1.05)
Married[i] living with children	1439 (36.0)	1	1	1
Single[j] living without children	1812 (33.6)	0.89 (0.83-0.96)	0.89 (0.83-0.96)	0.89 (0.83-0.96)

For peer review only - http://bmjopen.bmj.com/site/about/guidelines.xhtml

Single[j] living with children	277 (34.5)	0.96 (0.84-1.09)	0.96 (0.84-1.09)	0.96 (0.84-1.10)
**Labour market marginalisation characteristics**				
Trajectory groups of SA/DP from three years before up to inclusion				
Low increasing	2827 (35.1)	1	1	1
Low constant	951 (35.0)	0.96 (0.89-1.04)	0.96 (0.89-1.04)	0.97 (0.90-1.04)
Middle increasing	501 (35.3)	0.95 (0.87-1.06)	0.96 (0.87-1.05)	0.99 (0.89-1.09)
High decreasing	295 (37.2)	1.02 (0.90-1.16)	1.02 (0.90-1.16)	1.08 (0.95-1.23)
High constant	736 (35.2)	1.00 (0.92-1.09)	1.00 (0.91-1.09)	1.06 (0.97-1.17)
Unemployment in the year before inclusion				
No unemployment	4850 (35.2)	1	1	1
1-180 days	310 (36.4)	1.07 (0.95-1.20)	1.07 (0.95-1.20)	1.06 (0.94-1.19)
>180 days	150 (35.9)	1.08 (0.92-1.27)	1.08 (0.92-1.27)	1.07 (0.91-1.26)
**AMI-related characteristics**				
Type of infarction[k] at inclusion				
STEMI[l]	1945 (37.0)	1.19 (1.12-1.27)	1.19 (1.12-1.27)	1.14 (1.07-1.21)
Non-STEMI[m]	2311 (34.5)	1	1	1
Unspecified	1054 (34.0)	1.02 (0.95-1.10)	1.02 (0.95-1.10)	1.05 (0.98-1.13)
Coronary revascularisation characteristics[n] at inclusion				
Percutaneous coronary intervention	3772 (36.4)	1	1	1
Coronary artery bypass grafting	267 (79.5)	2.31 (2.04-2.62)	2.32 (2.05-2.63)	2.41 (2.13-2.74)
Others	1271 (29.1)	0.71 (0.67-0.76)	0.71 (0.67-0.76)	0.74 (0.69-0.79)
**Co-morbidity characteristics[k]**				
**Somatic co-morbidities[o] from three years before up to inclusion**				
Musculoskeletal disorders	835 (36.3)	1.04 (0.96-1.12)	1.04 (0.96-1.12)	1.05 (0.98-1.14)
Diabetes mellitus[p]	827 (32.7)	0.87 (0.81-0.94)	0.87 (0.81-0.94)	0.91 (0.84-0.98)

For peer review only

For peer review only - http://bmjopen.bmj.com/site/about/guidelines.xhtml

Risk factors for re-infarction and mortality after acute myocardial infarction

Renal insufficiency	73 (29.4)	0.74 (0.58-0.93)	0.74 (0.59-0.94)	0.84 (0.66-1.06)
Hypertension	1741 (34.1)	0.92 (0.87-0.98)	0.93 (0.87-0.98)	0.95 (0.89-1.00)
Stroke	59 (29.7)	0.76 (0.59-0.99)	0.77 (0.59-0.99)	0.81 (0.63-1.05)
Cancer	300 (32.2)	0.89 (0.79-1.00)	0.90 (0.80-1.01)	0.94 (0.83-1.06)
Other somatic disorders	3507 (34.7)	0.92 (0.87-0.97)	0.92 (0.87-0.97)	0.95 (0.90-1.01)
**Mental co-morbidities**				
Common mental disorders[o] from three years before up to inclusion	300 (37.9)	1.12 (1.00-1.27)	–	1.13 (1.00-1.28)
Other mental disorders[o] from three years before up to inclusion	474 (35.6)	1.06 (0.96-1.17)	–	1.05 (0.96-1.16)
Psychiatric medication[q] in the year before inclusion	1101 (34.1)	0.96 (0.90-1.04)	–	1.00 (0.93-1.07)

[a] Adjusted for sex, age, educational level, country of birth, type of living area, family situation, trajectory groups of SA/DP and previous unemployment
[b] Adjusted for sex, age, educational level, country of birth, type of living area, family situation, trajectory groups of SA/DP and previous unemployment, inpatient and specialised outpatient care due to common mental disorders and other mental disorders, and psychiatric medications; Mental co-morbidities were not mutually controlled
[c] Adjusted for sex, age, educational level, country of birth, type of living area, family situation, trajectory groups of SA/DP and previous unemployment, inpatient and specialised outpatient care due to common mental disorders and other mental disorders, and psychiatric medications, type of infarction, type of coronary revascularisation, musculoskeletal disorders, diabetes mellitus, renal insufficiency, hypertension, stroke, cancer and other somatic disorders; Mental co-morbidities were not mutually controlled
[d] Measured on 31st December of the year preceding acute myocardial infarction
[e] Missing data is considered compulsory education
[f] Missing data is considered Non-European countries
[g] Type of living area: big cities (Stockholm, Gothenburg and Malmö); medium sized cities (cities with more than 90 000 inhabitants within 30 km distance from the centre of the city); small cities/villages/rural
[h] Missing data is considered single living without children
[i] Married includes all living with partner; cohabitant
[j] Single includes divorced, separated, or widowed
[k] See method section for the International Classification of Diseases version 10 (ICD-10) codes or the Anatomic Therapeutic Chemical classification system (ATC) codes
[l] ST-elevation myocardial infarction
[m] Non-ST-elevation myocardial infarction
[n] See method section for the Classification of Surgical Procedures
[o] Measured by main or side diagnosis in inpatient or specialised outpatient care
[p] Additionally measured by prescribed antidiabetic medication
[q] Measured by antidepressants, anxiolytics and sedatives

For peer review only - http://bmjopen.bmj.com/site/about/guidelines.xhtml

Risk factors for re-infarction and mortality after acute myocardial infarction

Table 3. Adjusted hazard ratios (HR) and 95% Confidence Interval (CI) for all-cause mortality in individuals with a diagnosis of acute myocardial infarction (AMI) from inpatient care in 2008-2010 in Sweden (N=15 069) during the first year after AMI

Characteristics of patients with AMI	**Mortality**	Model 1[a]	Model 2[b]	Model 3[c]
	n (%)	HR (95% CI)		
**Socio-demographic characteristics[d]**				
Sex				
Men	475 (4.2)	1	1	1
Women	191 (5.2)	0.95 (0.80-1.14)	0.92 (0.78-1.10)	0.94 (0.78-1.12)
Age				
25-45	35 (2.5)	1	1	1
46-55	137 (2.9)	0.98 (0.67-1.42)	0.97 (0.66-1.40)	1.04 (0.71-1.51)
56-64	494 (5.5)	1.76 (1.23-2.50)	1.74 (1.22-2.48)	1.82 (1.27-2.60)
Education (years)[e]				
Compulsory (≤9)	255 (5.7)	1.28 (1.02-1.62)	1.29 (1.02-1.63)	1.29 (1.02-1.62)
High school (10–12)	305 (4.1)	1.06 (0.85-1.33)	1.06 (0.84-1.32)	1.05 (0.84-1.31)
University (>12)	106 (3.4)	1	1	1
Country of birth[f]				
Sweden	550 (4.6)	1	1	1
Other Nordic countries	52 (6.1)	1.02 (0.77-1.36)	1.03 (0.78-1.38)	1.22 (0.91-1.62)
Europe (except Nordic countries)	16 (3.6)	0.70 (0.42-1.15)	0.73 (0.44-1.20)	0.80 (0.48-1.32)
Non-European countries	48 (2.9)	0.58 (0.43-0.80)	0.59 (0.44-0.81)	0.62 (0.45-0.84)
Type of living area[g]				
Big cities	214 (4.7)	1	1	1
Medium sized cities	224 (4.2)	0.84 (0.69-1.02)	0.84 (0.69-1.01)	0.88 (0.72-1.06)
Small towns/villages	228 (4.4)	0.84 (0.69-1.01)	0.85 (0.70-1.03)	0.86 (0.71-1.05)
Family situation[h]				
Married[i] living without children	208 (4.3)	1.31 (1.02-1.70)	1.30 (1.01-1.68)	1.36 (1.05-1.76)
Married[i] living with children	94 (2.4)	1	1	1
Single[j] living without children	334 (6.2)	1.75 (1.38-2.22)	1.69 (1.33-2.14)	1.73 (1.36-2.20)

For peer review only - http://bmjopen.bmj.com/site/about/guidelines.xhtml

Risk factors for re-infarction and mortality after acute myocardial infarction

Single[j] living with children	30 (3.7)	1.28 (0.84-1.94)	1.25 (0.83-1.90)	1.28 (0.84-1.94)
**Labour market marginalisation characteristics**				
Trajectory groups of SA/DP from three years before up to inclusion				
Low increasing	243 (3.0)	1	1	1
Low constant	69 (2.5)	0.82 (0.63-1.07)	0.79 (0.60-1.03)	0.68 (0.52-0.89)
Middle increasing	71 (5.0)	1.60 (1.22-2.09)	1.47 (1.12-1.93)	1.02 (0.78-1.35)
High decreasing	18 (2.3)	0.59 (0.36-0.95)	0.52 (0.32-0.85)	0.33 (0.20-0.54)
High constant	265 (12.7)	3.94 (3.26-4.76)	3.45 (2.81-4.23)	2.16 (1.75-2.70)
Unemployment in the year before inclusion				
No unemployment	622 (4.5)	1	1	1
1-180 days	27 (3.2)	1.07 (0.72-1.59)	1.05 (0.71-1.56)	1.04 (0.70-1.55)
>180 days	17 (4.1)	1.31 (0.81-2.14)	1.31 (0.81-2.14)	1.28 (0.79-2.09)
**AMI-related characteristics**				
Type of infarction[k] at inclusion				
STEMI[l]	267 (5.1)	2.45 (2.01-2.99)	2.48 (2.03-3.03)	3.80 (3.08-4.68)
Non-STEMI[m]	153 (2.3)	1	1	1
Unspecified	246 (7.9)	3.45 (2.82-4.23)	3.45 (2.82-4.22)	2.97 (2.42-3.65)
Coronary revascularisation characteristics[n] at inclusion				
Percutaneous coronary intervention	257 (2.5)	1	1	1
Coronary artery bypass grafting	11 (3.3)	1.26 (0.69-2.31)	1.27 (0.70-2.33)	1.65 (0.90-3.02)
Others	398 (9.1)	3.30 (2.81-3.87)	3.25 (2.76-3.81)	3.60 (3.03-4.28)
**Co-morbidity characteristics[k]**				
**Somatic co-morbidities[o] from three years before up to inclusion**				
Musculoskeletal disorders	112 (4.9)	0.90 (0.73-1.11)	0.91 (0.74-1.12)	1.00 (0.81-1.24)
Diabetes mellitus[p]	199 (7.9)	1.73 (1.46-2.05)	1.74 (1.47-2.06)	1.70 (1.42-2.03)
Renal insufficiency	60 (24.2)	4.34 (3.31-5.71)	4.29 (3.26-5.64)	2.59 (1.95-3.45)

For peer review only - http://bmjopen.bmj.com/site/about/guidelines.xhtml

Hypertension	198 (3.9)	0.74 (0.62-0.87)	0.74 (0.62-0.87)	0.68 (0.57-0.81)
Stroke	25 (12.6)	1.96 (1.31-2.94)	1.99 (1.33-2.98)	1.63 (1.09-2.45)
Cancer	102 (10.9)	2.46 (1.99-3.05)	2.45 (1.98-3.04)	2.22 (1.78-2.75)
Other somatic disorders	530 (5.2)	1.62 (1.34-1.96)	1.59 (1.31-1.92)	1.46 (1.20-1.78)
**Mental co-morbidities**				
Common mental disorders[o] from three years before up to inclusion	43 (5.4)	0.80 (0.59-1.11)	–	0.90 (0.66-1.24)
Other mental disorders[o] from three years before up to inclusion	101 (7.6)	1.40 (1.13-1.74)	–	1.46 (1.17-1.82)
Psychiatric medication[q] in the year before inclusion	241 (7.5)	1.39 (1.16-1.66)	–	1.24 (1.03-1.48)

[a] Adjusted for sex, age, educational level, country of birth, type of living area, family situation, trajectory groups of SA/DP and previous unemployment
[b] Adjusted for sex, age, educational level, country of birth, type of living area, family situation, trajectory groups of SA/DP and previous unemployment, inpatient and specialised outpatient care due to common mental disorders and other mental disorders, and psychiatric medications; Mental co-morbidities were not mutually controlled
[c] Adjusted for sex, age, educational level, country of birth, type of living area, family situation, trajectory groups of SA/DP and previous unemployment, inpatient and specialised outpatient care due to common mental disorders and other mental disorders, and psychiatric medications, type of infarction, type of coronary revascularisation, musculoskeletal disorders, diabetes mellitus, renal insufficiency, hypertension, stroke, cancer and other somatic disorders; Mental co-morbidities were not mutually controlled
[d] Measured on 31st December of the year preceding acute myocardial infarction
[e] Missing data is considered compulsory education
[f] Missing data is considered Non-European countries
[g] Type of living area: big cities (Stockholm, Gothenburg and Malmö); medium sized cities (cities with more than 90 000 inhabitants within 30 km distance from the centre of the city); small cities/villages/rural
[h] Missing data is considered single living without children
[i] Married includes all living with partner; cohabitant
[j] Single includes divorced, separated, or widowed
[k] See method section for the International Classification of Diseases version 10 (ICD-10) codes or the Anatomic Therapeutic Chemical classification system (ATC) codes
[l] ST-elevation myocardial infarction
[m] Non-ST-elevation myocardial infarction
[n] See method section for the Classification of Surgical Procedures
[o] Measured by main or side diagnosis in inpatient or specialised outpatient care
[p] Additionally measured by prescribed antidiabetic medication
[q] Measured by antidepressants, anxiolytics and sedatives

For peer review only - http://bmjopen.bmj.com/site/about/guidelines.xhtml

Risk factors for re-infarction and mortality after acute myocardial infarction

Figure 1. Trajectory groups of sickness absence and disability pension (SA/DP) months before the hospital admission date for acute myocardial infarction (AMI) in 2008-2010 (T0) and percentages of individuals in each trajectory group (n=15 069). The dotted lines represent 95% CIs.

For peer review only

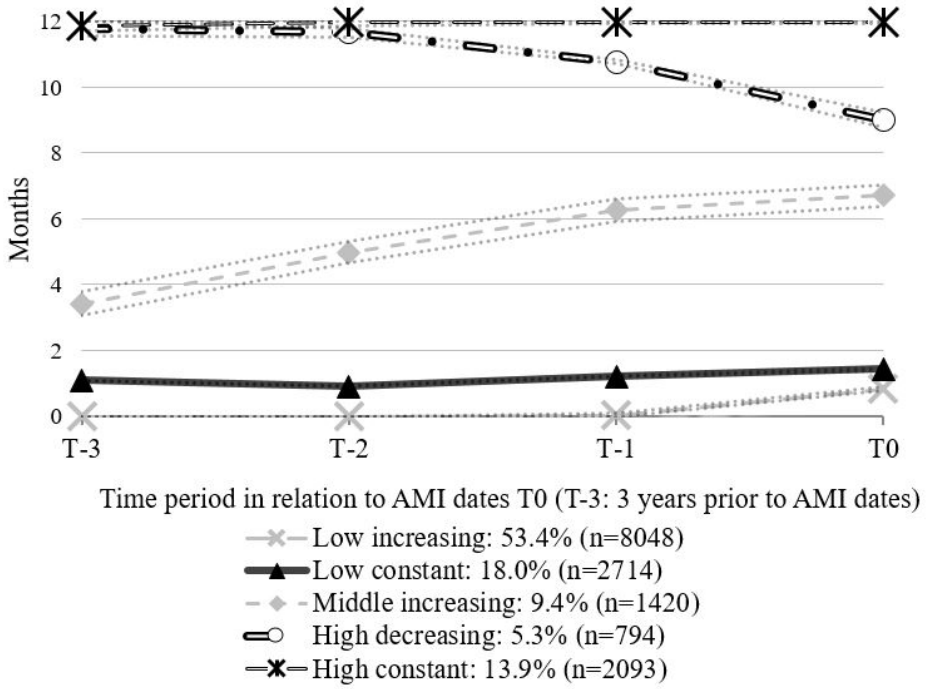

Figure 1. Trajectory groups of sickness absence and disability pension (SA/DP) months before the hospital admission date for acute myocardial infarction (AMI) in 2008-2010 (T0) and percentages of individuals in each trajectory group (n=15 069). The dotted lines represent 95% CIs.

211x150mm (96 x 96 DPI)

Supplementary Table

Table. Crude and adjusted hazard ratios (HR) and 95% Confidence Interval (CI) for mortality due to circulatory disorders during first year of follow-up in individuals with diagnosis of acute myocardial infarction (AMI) from inpatient care in 2008-2010 in Sweden (N=15 069)

Characteristics of patients with AMI	**Mortality (First year)** n (%)	Crude model	Model 1[a]	Model 2[b]	Model 3[c]
		HR (95% CI)			
**Socio-demographic characteristics[d]**					
Sex					
Men	365 (3.1)	1	1	1	1
Women	133 (3.6)	1.14 (0.94-1.40)	0.92 (0.75-1.13)	0.91 (0.74-1.12)	0.93 (0.75-1.15)
Age					
25-45	24 (1.7)	1	1	1	1
46-55	99 (2.1)	1.21 (0.77-1.89)	1.04 (0.66-1.63)	1.02 (0.65-1.60)	1.15 (0.73-1.81)
56-64	375 (4.1)	2.40 (1.59-3.63)	1.89 (1.24-2.90)	1.85 (1.21-2.84)	2.10 (1.37-3.23)
Education (years)[e]					
Compulsory (≤9)	190 (4.1)	1.61 (1.25-2.09)	1.26 (0.96-1.64)	1.25 (0.96-1.63)	1.23 (0.94-1.61)
High school (10–12)	225 (3.0)	1.16 (0.90-1.49)	1.02 (0.79-1.32)	1.02 (0.79-1.31)	1.02 (0.79-1.32)
University (>12)	83 (2.6)	1	1	1	1
Country of birth[f]					
Sweden	408 (3.3)	1	1	1	1
Other Nordic countries	44 (5.0)	1.51 (1.11-2.07)	1.20 (0.87-1.64)	1.20 (0.87-1.64)	1.32 (0.97-1.81)
Europe (except Nordic countries)	11 (2.5)	0.73 (0.40-1.32)	0.69 (0.38-1.25)	0.70 (0.38-1.28)	0.76 (0.42-1.39)
Non-European countries	35 (2.1)	0.61 (0.43-0.87)	0.62 (0.43-0.89)	0.63 (0.44-0.90)	0.67 (0.46-0.96)
Type of living area[g]					
Big cities	150 (3.2)	1	1	1	1
Medium sized cities	173 (3.2)	0.99 (0.79-1.23)	0.92 (0.74-1.15)	0.93 (0.75-1.17)	0.97 (0.78-1.21)
Small towns/villages	175 (3.3)	1.04 (0.83-1.29)	0.92 (0.73-1.15)	0.94 (0.75-1.18)	0.93 (0.74-1.16)
Family situation[h]					
Married[i] living without children	172 (3.4)	1.95 (1.48-2.57)	1.41 (1.06-1.88)	1.40 (1.05-1.87)	1.47 (1.10-1.97)

For peer review only

Married[i] living with children	72 (1.8)	1	1	1	1
Single[j] living without children	231 (4.2)	2.40 (1.84-3.12)	1.64 (1.24-2.15)	1.59 (1.20-2.09)	1.61 (1.22-2.12)
Single[j] living with children	23 (2.8)	1.60 (1.00-2.56)	1.36 (0.84-2.18)	1.33 (0.83-2.15)	1.37 (0.85-2.20)
**Work-related characteristics**					
Trajectory groups of SA/DP* three years before and at inclusion					
Low increasing	210 (2.6)	1	1	1	1
Constant low	47 (1.7)	0.66 (0.48-0.90)	0.65 (0.47-0.89)	0.64 (0.46-0.88)	0.58 (0.42-0.80)
Middle increasing	47 (3.2)	1.25 (0.91-1.72)	1.22 (0.89-1.68)	1.17 (0.85-1.62)	0.93 (0.67-1.29)
High decreasing	13 (1.5)	0.60 (0.34-1.05)	0.47 (0.26-0.82)	0.44 (0.25-0.77)	0.33 (0.18-0.58)
Constant high	181 (8.3)	3.29 (2.69-4.01)	3.06 (2.47-3.80)	2.82 (2.23-3.57)	1.97 (1.54-2.53)
Unemployment in the year before inclusion					
No unemployment	462 (3.3)	1	1	1	1
1-180 days	18 (2.1)	0.63 (0.39-1.01)	0.95 (0.59-1.54)	0.94 (0.58-1.52)	0.94 (0.58-1.52)
>180 days	18 (4.3)	1.30 (0.81-2.08)	1.82 (1.13-2.93)	1.83 (1.14-2.96)	1.83 (1.13-2.97)
**AMI characteristics**					
Inpatient care due to AMI (median 4 days)[k] at inclusion					
1-4 days	316 (3.3)	1	1	1	1
>4 days	182 (3.2)	0.95 (0.79-1.14)	0.86 (0.72-1.04)	0.86 (0.71-1.03)	0.74 (0.61-0.89)
Type of infarction[k] at inclusion					
STEMI[l]	219 (4.1)	2.78 (2.20-3.51)	2.98 (2.35-3.76)	3.00 (2.37-3.80)	4.29 (3.36-5.48)
Non-STEMI[m]	104 (1.5)	1	1	1	1
Unspecified	175 (5.5)	3.73 (2.93-4.75)	3.62 (2.84-4.61)	3.58 (2.81-4.57)	3.13 (2.45-4.00)
Coronary revascularisation characteristics[n] at inclusion					
Percutaneous coronary intervention	211 (2.0)	1	1	1	1
Coronary artery bypass grafting	10 (2.9)	1.47 (0.78-2.78)	1.36 (0.72-2.57)	1.38 (0.73-2.60)	1.88 (0.99-3.56)
Others	277 (6.1)	3.12 (2.61-3.74)	2.80 (2.33-3.36)	2.76 (2.29-3.32)	3.37 (2.76-4.11)

For peer review only

**Morbidity characteristics**					
**Somatic (co)-morbidities[o] three years before and/or at inclusion**					
Musculoskeletal disorders					
No	423 (3.3)	1	1	1	1
Yes	75 (3.2)	0.98 (0.77-1.26)	0.84 (0.66-1.08)	0.84 (0.66-1.08)	0.91 (0.71-1.18)
Diabetes mellitus[p]					
No	360 (2.8)	1	1	1	1
Yes	138 (5.2)	1.87 (1.53-2.27)	1.55 (1.27-1.89)	1.56 (1.27-1.91)	1.53 (1.24-1.90)
Renal insufficiency					
No	458 (3.0)	1	1	1	1
Yes	40 (15.4)	5.29 (3.83-7.30)	3.88 (2.78-5.40)	3.75 (2.68-5.25)	2.75 (1.94-3.89)
Hypertension					
No	353 (3.5)	1	1	1	1
Yes	145 (2.8)	0.79 (0.65-0.96)	0.71 (0.59-0.87)	0.71 (0.59-0.87)	0.67 (0.55-0.82)
Stroke					
No	477 (3.1)	1	1	1	1
Yes	21 (10.1)	3.33 (2.15-5.15)	2.26 (1.45-3.51)	2.30 (1.48-3.58)	1.96 (1.25-3.06)
Cancer					
No	459 (3.2)	1	1	1	1
Yes	39 (4.1)	1.28 (0.93-1.78)	1.15 (0.83-1.60)	1.13 (0.81-1.57)	1.01 (0.72-1.41)
Other somatic disorders					
No	115 (2.3)	1	1	1	1
Yes	383 (3.7)	1.62 (1.32-2.00)	1.43 (1.16-1.77)	1.41 (1.14-1.75)	1.36 (1.10-1.70)
**Mental co-morbidities**					
Common mental disorders[o] three years before and/or at inclusion					
No	471 (3.2)	1	1	–	1
Yes	27 (3.4)	1.04 (0.70-1.53)	0.76 (0.51-1.13)	–	0.81 (0.54-1.21)
Other mental disorders[o]					

For peer review only

three years before and/or at inclusion					
No	431 (3.1)	1	1	–	1
Yes	67 (5.0)	1.63 (1.26-2.11)	1.31 (1.01-1.71)	–	1.28 (0.98-1.68)
Antidepressants					
in the year before inclusion					
No antidepressants	418 (3.1)	1	1	–	1
Small doses (<0.5 DDD[q])	24 (4.0)	1.26 (0.84-1.91)	1.05 (0.69-1.59)	–	0.96 (0.63-1.46)
Moderate doses (0.5-1.5 DDD)	42 (4.8)	1.57 (1.14-2.16)	1.15 (0.83-1.59)	–	1.17 (0.84-1.63)
High doses (>1.5 DDD)	14 (3.4)	1.12 (0.66-1.90)	0.70 (0.41-1.21)	–	0.81 (0.47-1.40)
Anxiolytics					
in the year before inclusion					
No Anxiolytics	431 (3.1)	1	1	–	1
Small doses (<0.5 DDD)	45 (4.5)	1.48 (1.09-2.01)	1.10 (0.80-1.51)	–	1.06 (0.77-1.46)
Moderate doses (0.5-1.5 DDD)	13 (6.1)	1.98 (1.14-3.44)	1.10 (0.63-1.93)	–	1.04 (0.59-1.83)
High doses (>1.5 DDD)	<10 (8.0)	2.63 (1.36-5.09)	1.15 (0.59-2.25)	–	1.22 (0.62-2.40)
Sedatives					
in the year before inclusion					
No sedatives	394 (2.9)	1	1	–	1
Small doses (<0.5 DDD)	41 (4.5)	1.58 (1.14-2.17)	1.33 (0.96-1.85)	–	1.16 (0.83-1.61)
Moderate doses (0.5-1.5 DDD)	40 (6.5)	2.25 (1.63-3.12)	1.42 (1.01-1.99)	–	1.28 (0.90-1.80)
High doses (>1.5 DDD)	23 (7.5)	2.65 (1.74-4.03)	1.52 (0.98-2.36)	–	1.47 (0.95-2.28)

[a] Adjusted for sex, age, educational level, country of birth, type of living area, family situation, trajectory groups of SA/DP and previous unemployment

[b] Adjusted for sex, age, educational level, country of birth, type of living area, family situation, trajectory groups of SA/DP and previous unemployment, in- and specialised outpatient care due to common mental disorders and other mental disorders, antidepressants, anxiolytics and sedatives; Mental comorbidities were not mutually controlled

[c] Adjusted for sex, age, educational level, country of birth, type of living area, family situation, trajectory groups of SA/DP and previous unemployment, in- and specialised outpatient care due to common mental disorders and other mental disorders, antidepressants, anxiolytics, sedatives, inpatient days due to AMI, type of infarction, musculoskeletal disorders, diabetes mellitus, renal insufficiency, hypertension, stroke, cancer and other somatic disorders; Mental comorbidities were not mutually controlled

[d] Measured on 31st December of the year preceding acute myocardial infarction

For peer review only

[e] Missing data is considered compulsory education
[f] Missing data is considered Non-European countries
[g] Type of living area: big cities (Stockholm, Gothenburg and Malmö); medium sized cities (cities with more than 90 000 inhabitants within 30 km distance from the centre of the city); small cities/villages/rural
[h] Missing data is considered single living without children
[i] Married includes all living with partner; cohabitant
[j] Single includes divorced, separated, or widowed
[k] See method section for the International Classification of Diseases version 10 (ICD-10) codes or the Anatomic Therapeutic Chemical classification system (ATC) codes
[l] ST-elevation myocardial infarction
[m] Non-ST-elevation myocardial infarction
[n] See method section for the Classification of Surgical Procedures
[o] Measured by main or side diagnosis in in- or specialised outpatient care
[p] Additionally measured by prescribed antidiabetic medication
[q] Daily dispensed dose (DDD)

For peer review only

Reporting checklist for cohort study.

Based on the STROBE cohort guidelines.

Instructions to authors

Complete this checklist by entering the page numbers from your manuscript where readers will find each of the items listed below.

Your article may not currently address all the items on the checklist. Please modify your text to include the missing information. If you are certain that an item does not apply, please write "n/a" and provide a short explanation.

Upload your completed checklist as an extra file when you submit to a journal.

In your methods section, say that you used the STROBE cohortreporting guidelines, and cite them as:

von Elm E, Altman DG, Egger M, Pocock SJ, Gotzsche PC, Vandenbroucke JP. The Strengthening the Reporting of Observational Studies in Epidemiology (STROBE) Statement: guidelines for reporting observational studies.

		Reporting Item	Page Number
**Title and abstract**			
Title	#1a	Indicate the study’s design with a commonly used term in the title or the abstract	1

Abstract	#1b	Provide in the abstract an informative and balanced summary of what was done and what was found	2-3
**Introduction**			
Background / rationale	#2	Explain the scientific background and rationale for the investigation being reported	5-6
Objectives	#3	State specific objectives, including any prespecified hypotheses	6
**Methods**			
Study design	#4	Present key elements of study design early in the paper	7
Setting	#5	Describe the setting, locations, and relevant dates, including periods of recruitment, exposure, follow-up, and data collection	7-8
Eligibility criteria	#6a	Give the eligibility criteria, and the sources and methods of selection of participants. Describe methods of follow-up.	7
Eligibility criteria	#6b	For matched studies, give matching criteria and number of exposed and unexposed	n/a It is not a matched study.
Variables	#7	Clearly define all outcomes, exposures, predictors, potential confounders, and effect modifiers. Give diagnostic criteria, if applicable	8-9

For peer review only

Data sources / measurement	#8	For each variable of interest give sources of data and details of methods of assessment (measurement). Describe comparability of assessment methods if there is more than one group. Give information separately for for exposed and unexposed groups if applicable.	7-9
Bias	#9	Describe any efforts to address potential sources of bias	17
Study size	#10	Explain how the study size was arrived at	7
Quantitative variables	#11	Explain how quantitative variables were handled in the analyses. If applicable, describe which groupings were chosen, and why	7-8
Statistical methods	#12a	Describe all statistical methods, including those used to control for confounding	8-9
Statistical methods	#12b	Describe any methods used to examine subgroups and interactions	8-9
Statistical methods	#12c	Explain how missing data were addressed	8-9
Statistical methods	#12d	If applicable, explain how loss to follow-up was addressed	n/a there is no loss to follow-up
Statistical methods	#12e	Describe any sensitivity analyses	9

Results

For peer review only

Participants	#13a	Report numbers of individuals at each stage of study—eg numbers potentially eligible, examined for eligibility, confirmed eligible, included in the study, completing follow-up, and analysed. Give information separately for for exposed and unexposed groups if applicable.	11
Participants	#13b	Give reasons for non-participation at each stage	n/a
Participants	#13c	Consider use of a flow diagram	n/a
Descriptive data	#14a	Give characteristics of study participants (eg demographic, clinical, social) and information on exposures and potential confounders. Give information separately for exposed and unexposed groups if applicable.	11
Descriptive data	#14b	Indicate number of participants with missing data for each variable of interest	n/a
Descriptive data	#14c	Summarise follow-up time (eg, average and total amount)	10
Outcome data	#15	Report numbers of outcome events or summary measures over time. Give information separately for exposed and unexposed groups if applicable.	11
Main results	#16a	Give unadjusted estimates and, if applicable, confounder-adjusted estimates and their precision (eg, 95% confidence interval). Make clear which confounders were adjusted for and why they were included	11-12

For peer review only

Main results	#16b	Report category boundaries when continuous variables were categorized	11-12
Main results	#16c	If relevant, consider translating estimates of relative risk into absolute risk for a meaningful time period	n/a
Other analyses	#17	Report other analyses done—e.g., analyses of subgroups and interactions, and sensitivity analyses	n/a
**Discussion**			
Key results	#18	Summarise key results with reference to study objectives	17
Limitations	#19	Discuss limitations of the study, taking into account sources of potential bias or imprecision. Discuss both direction and magnitude of any potential bias.	17
Interpretation	#20	Give a cautious overall interpretation considering objectives, limitations, multiplicity of analyses, results from similar studies, and other relevant evidence.	13-17
Generalisability	#21	Discuss the generalisability (external validity) of the study results	16
**Other Information**			
Funding	#22	Give the source of funding and the role of the funders for the present study and, if applicable, for the original study on which the present article is based	18

Notes:

- 6b: n/a It is not a matched study.

For peer review only

- 12d: n/a there is no loss to follow-up The STROBE checklist is distributed under the terms of the Creative Commons Attribution License CC-BY. This checklist was completed on 13. August 2019 using https://www.goodreports.org/, a tool made by the EQUATOR Network in collaboration with Penelope.ai

For peer review only
